# Depression and health outcomes: An umbrella review of systematic reviews and meta-analyses of observational studies

**DOI:** 10.1038/s41398-025-03463-8

**Published:** 2025-08-20

**Authors:** Xiaojuan Chen, Xiaoning Liu, Fengjuan Li, Haitian He, Xinying Li, Tianhang Qin, Bin Jiang, Yuge Chen, Yanqi Wang, Yuhao Su, Xiaojie Wang, Lei Liang, Huiling Hua, Jun Wu, Jianping Ma, Fulan Hu, Pei Qin

**Affiliations:** 1 School of Public Health, Shantou University, Shantou, Guangdong China; 2 Department of Respiratory and Critical Care Medicine, the Second Affiliated Hospital of Zhengzhou University, Zhengzhou, China; 3 Department of Urology, Shenzhen Qianhai Shekou Free Trade Zone Hospital, Shenzhen, China; 4 Institute of Software Chinese Academy of Sciences, Beijing, Guangdong China; 5 Department of Neurology, Shenzhen Qianhai Shekou Free Trade Zone Hospital, Shenzhen, Guangdong China; 6 Department of Gynecology and Obstetrics, Shenzhen Qianhai Shekou Free Trade Zone Hospital, Shenzhen, Guangdong China; 7 Department of Biostatistics and Epidemiology, School of Public Health, Shenzhen University Medical School, Shenzhen, China; 8 Department of Behavioural Science and Health, University College London, London, UK

**Keywords:** Depression, Diseases

## Abstract

**Background:**

Currently, most studies of depression are limited to a single disease endpoint.

**Aims:**

This study aimed to conduct an umbrella review to comprehensively assess the association between depression and health outcomes.

**Method:**

Until December 17, 2024, we conducted a systematic search of systematic reviews and meta-analyses in PubMed, Embase, and Web of Science. We reanalyzed the summary effects and 95% confidence intervals for each study using random models. We assessed the methodological quality and evidence quality of the research with A Measurement Tool to Assess Systematic Reviews 2 and Grade of Recommendations, Assessment, Development and Evaluation, classifying studies into four categories based on evidence classification criteria.

**Results:**

We selected a total of 72 articles from 27,150 resulting in 114 meta-analyses and 109 health outcomes. Depression exposure was associated with 23 mortality, 21 cardiovascular outcomes, 15 offspring outcomes, 9cancer outcomes, 9 neurological outcomes, 5 endocrine outcomes, 5 dental outcomes, 3 digestive outcomes, and 19 other health outcomes. Moderate-quality evidence linked depression to specific mortality in bladder cancer (Class IV), all-cause mortality in myocardial infarction (Class III), mortality within 2 years of initial assessment in coronary artery disease (Class IV), major adverse cardiovascular events after percutaneous coronary intervention (Class III), irritable bowel syndrome (insignificant), fear of falling (Class III), and frailty (Class III).

**Conclusions:**

Depression has a significant impact on health outcomes, primarily mortality and cardiovascular outcomes. However, more definitive conclusions still require randomized controlled trials or prospective studies for validation.

## Introduction

Depression affects approximately 5% of adults worldwide, as the World Health Organization estimates, and it stands as the fourth most common disease globally [1]. According to the Global Burden of Disease data from 2019, depression was one of the leading causes of disability and mortality [2]. In 2020, there was an addition of 76.2 million cases of Major Depressive Disorder (MDD) worldwide. Majority of countries such as Spain, Mexico, Malaysia, the United States, and Uruguay experienced the most significant increase in depression prevalence [3]. Furthermore, it was estimated that there would be an additional 53.2 million cases of severe depression globally, representing an increase of 27.6% [4].

Recently, increasing number of studies have shown that depression can lead to various health outcomes such as cardiovascular disease, atrial fibrillation, heart failure, coronary artery disease, myocardial infarction, stroke, and hypertension and impacts life quality and longevity [5–10]. Depression also increases the risk of mortality, including cardiovascular mortality and all-cause mortality in cancer [11]. As the amount of systematic reviews and meta-analyses accumulates over the past decades and an umbrella review is helpful to provide a broader picture of findings and synthesize the evidence, depression-related umbrella reviews are mostly restricted to mortality, and many relevant meta-analyses have been published subsequently [12–16], and a lack of comprehensive and systematic assessment of the relationship between depression and multiple health outcomes. Additionally, due to the subjectivity or inconsistency of assessment criteria, differing definitions of exposure, as well as limitations in data sources, diversity in study designs, and inconsistencies in statistical methods, the quality of evidence in these reviews varies. An umbrella review, which synthesizes existing systematic reviews and meta-analyses, will provide decision-makers with a comprehensive source of high-quality research on the relationship between depression and various health outcomes.

Therefore, using umbrella review, this study conducted a comprehensive overview to thoroughly outline and assess the association between depression and various health outcomes.

## Methods

### Umbrella review methods

We comprehensively and systematically searched the existing literature for various systematic reviews and meta-analyses on the relationship between depression and health outcomes. This umbrella review was registered with the International Prospective Register of Systematic Reviews (PROSPERO) (CRD42023471844) (https://www.crd.york.ac.uk/PROSPERO/).

### Literature search

We systematically searched for systematic reviews and meta-analyses of observational studies in PubMed, Embase, and Web of Science databases from the database inception to December 17, 2024 (see Additional File 1: Table S1) written in English. Electronic searches were independently conducted by two authors (XC and PQ). Subsequently, duplicates were removed, titles and abstracts were screened, and full texts were read to identify meta-analyses that met the inclusion criteria. Any discrepancies between the two reviewers during the literature screening process were resolved by a third author. We manually searched the reference lists of all included articles, reviews, and meta-analyses to identify any potentially missed studies.

### Eligibility criteria

We included studies that met the following PICOS criteria: (1) population: participants without restrictions based on race, region, or health status; (2) intervention/exposure: with depression; (3) comparison: without depression;(4) outcome: any health outcome, defined as health states or results that affect individuals’ physical, psychological, or social functioning; and (5) study design: systematic reviews and meta-analyses of observational epidemiological studies (cohort, case-control, and cross-sectional studies) or randomized controlled trials.

We excluded articles that met any of the following criteria: (1) did not report summary estimates (e.g., systematic reviews without meta-analyses); (2) reported other mental disorders (such as anxiety) unless separate data on depression, as defined above; (3) were letters, conference abstracts, academic papers, and research protocols; (4) involved animal or cell culture research; and (5) were published not in English. If two or more health outcomes were reported in one article, data for each individual outcome were extracted separately. If studies on depression exposure and the same health outcome were published more than 24 months apart, we included the study with the newest data, usually the one with the largest sample size.

### Data extraction

Two researchers (XC and PQ) independently extracted the following data from eligible original articles: the first author’s name; publication date; study population; types of studies included in the meta-analysis (randomized controlled trials, cross-sectional, cohort, or case-control studies); exposure types; outcomes and the number of studies, total number of participants, and the number of cases included in the meta-analysis. Furthermore, the reviewers extracted the summary effect size and 95% confidence interval (CI) of the results, as well as the model for effect (random or fixed), heterogeneity (I^2^ statistic and Cochran Q test *P*-value), and publication bias assessment (Egger’s test or *P*-value of the funnel plot). Any disagreement was determined by a third author.

### Quality assessment of methods and evidence

A Measurement Tool to Assess Systematic Reviews 2 (AMSTAR2) was utilized to assess the methodological quality of the included articles [17]. AMSTAR2 is an effective and reliable measurement tool for evaluating the quality of systematic reviews and meta-analyses, categorizing study quality into four levels: “High”, “Moderate”, “Low” and “Critically low”. Simultaneously, Grading of Recommendations, Assessment, Development, and Evaluation (GRADE) was employed to assess the quality of evidence for the association between depression and each health outcome, categorized into four levels: “High”, “Moderate”, “Low”, or “Very low” [18]. Additionally, we classified the evidence into four categories based on evidence classification criteria: Class I (convincing evidence), Class II (highly suggestive evidence), Class III (suggestive evidence), Class IV (weak evidence), and Class NS (not significant) [19].

### Data analysis

We reanalyzed the summary effects (risk ratio, odds ratio, weighted mean difference, or standardized mean difference) for each study using a random-effects or fixed-effects model, and also recalculated their 95% confidence intervals. The I^2^ statistic and *P* value of Cochran’s Q test for heterogeneity were recalculated. Additionally, we employed Egger’s regression test to calculate estimates of publication bias for any reanalysis involving at least 10 studies, considering a *P*-value < 0.1 as significant [20]. If reanalysis could not be performed from the meta-analysis, we extracted summary data to evaluate heterogeneity and publication bias. We conducted the reanalysis with Stata MP version 17 and constructed summary forest plots based on the extracted and/or reanalyzed data using R version 4.3.2.

### Patient involvement

No patients participated in the planning, design, or implementation of this study.

## Results

### Characteristics of meta-analyses

**Fig. 1:**
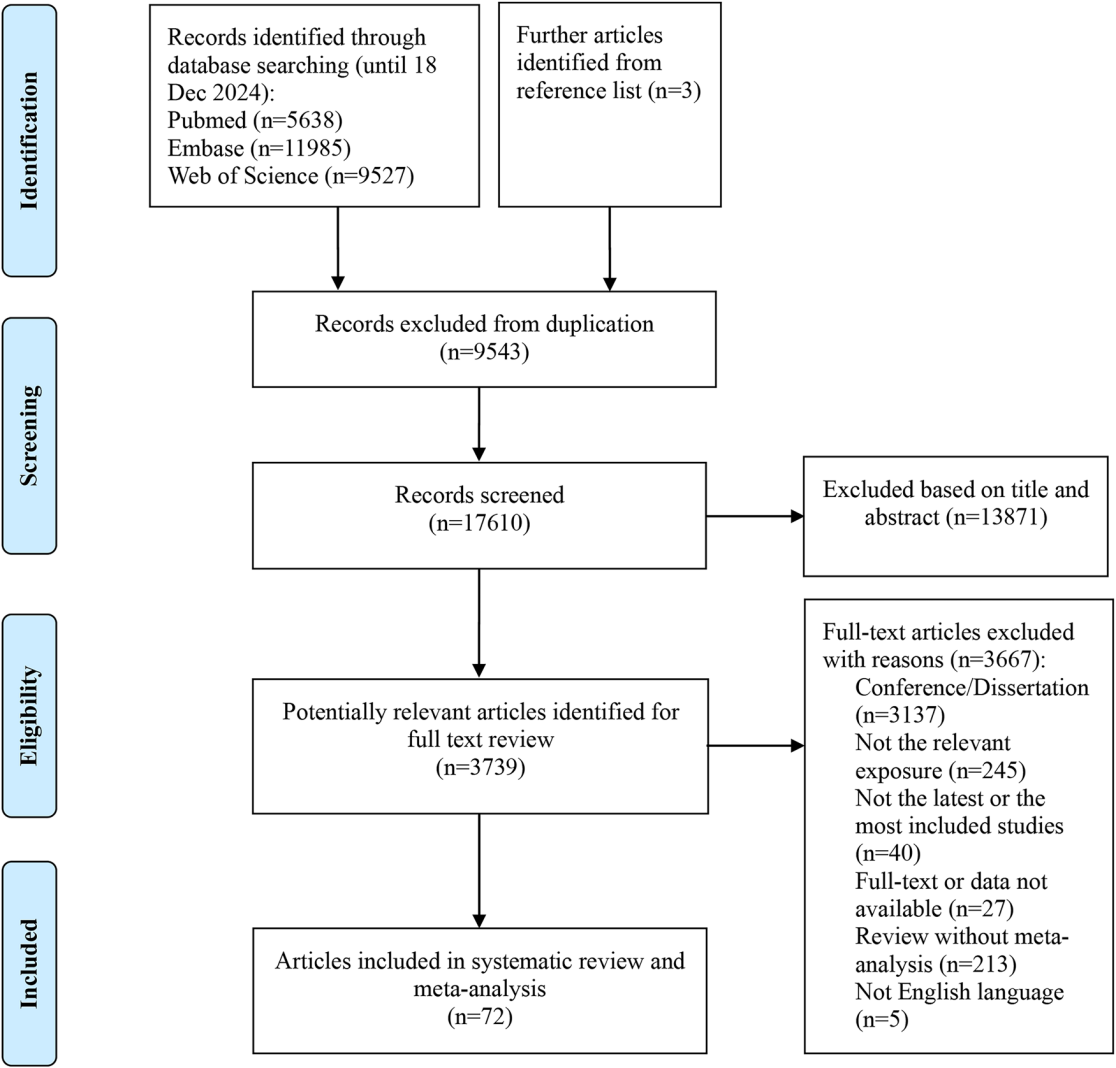
Flow chart of study selection.

**Fig. 2:**
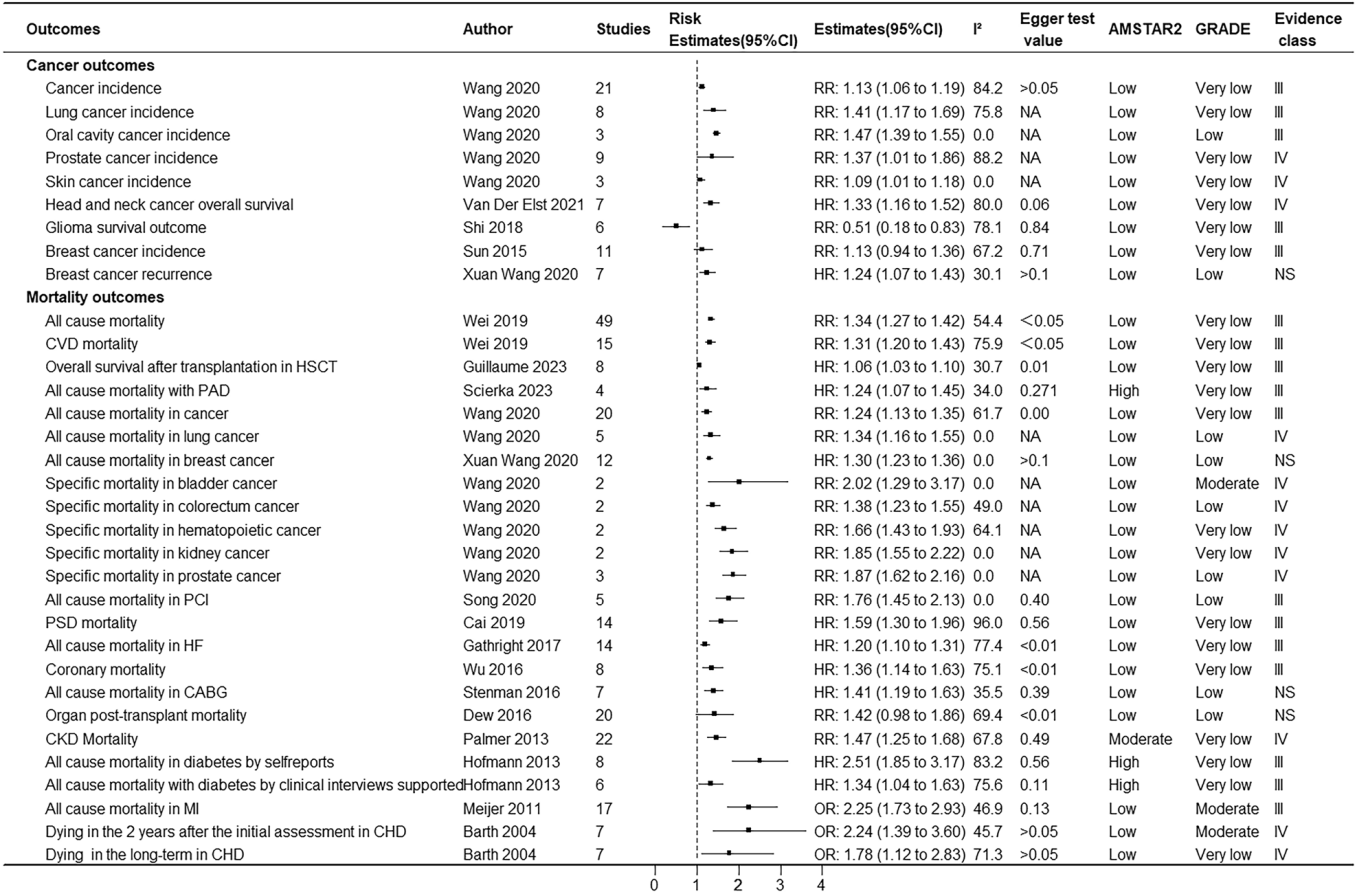
Associations between depression and cancer and mortality. NA not available, RR relative risk, OR odds ratio, HR hazard ratio, AMSTAR2 a measurement tool to assess systematic reviews 2, GRADE grading of recommendations, assessment, development, and evaluation, CVD cardiovascular diseases, HSCT hematopoietic stem cell transplantation, PAD peripheral artery disease, PCI percutaneous coronary intervention, PSD post-stroke depression, HF heart failure, CABG coronary artery bypass grafting, CKD chronic kidney disease, MI myocardial infarction, CHD coronary heart disease.

**Fig. 3:**
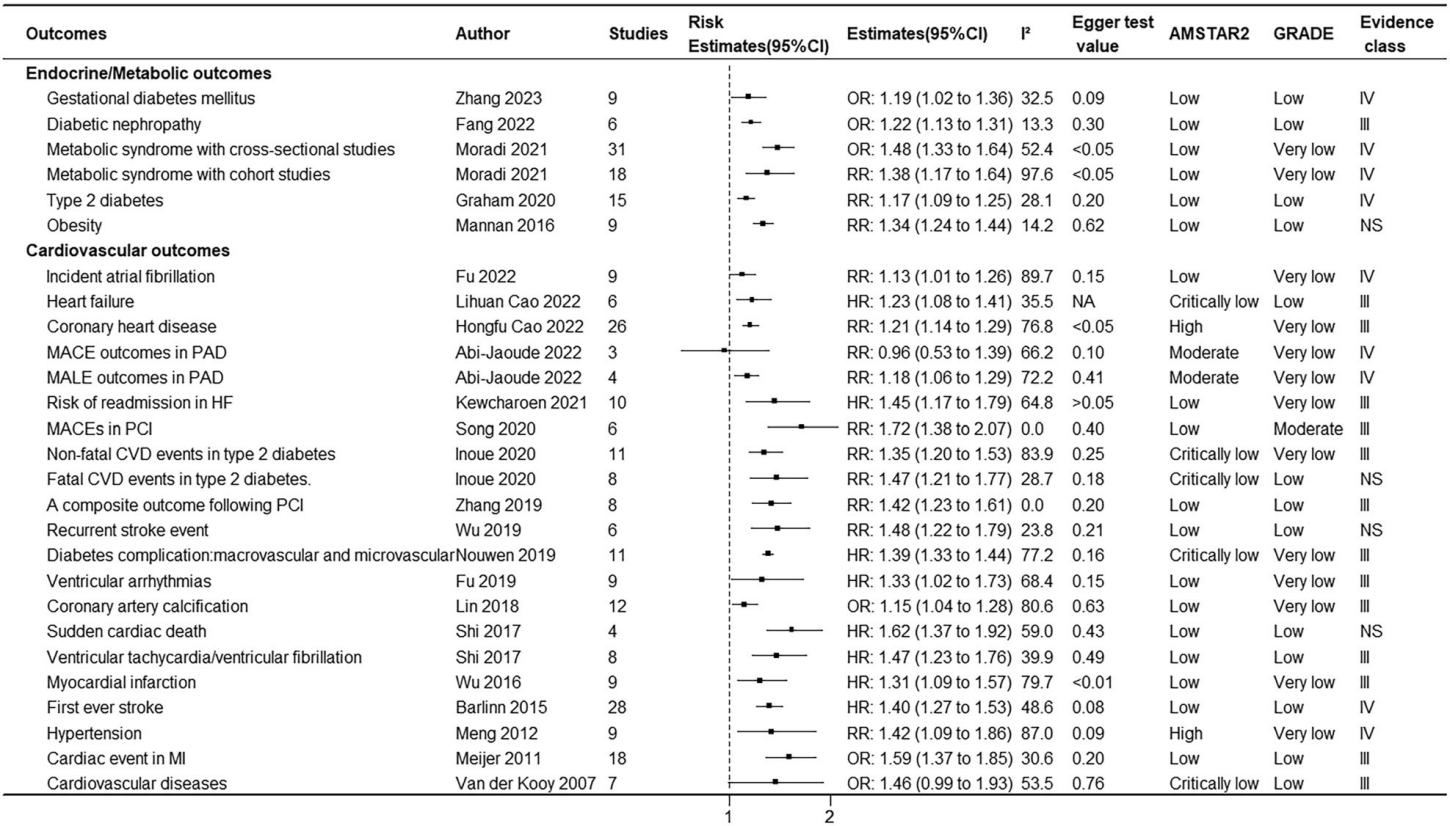
Associations between depression and endocrine/metabolic and cardiovascular outcomes. NA not available, RR relative risk, OR odds ratio, HR hazard ratio, AMSTAR2 a measurement tool to assess systematic reviews 2, GRADE Grading of Recommendations, Assessment, Development, and Evaluation, PAD peripheral artery disease, HF heart failure, PCI percutaneous coronary intervention, CVD cardiovascular diseases, MI myocardial infarction.

**Fig. 4:**
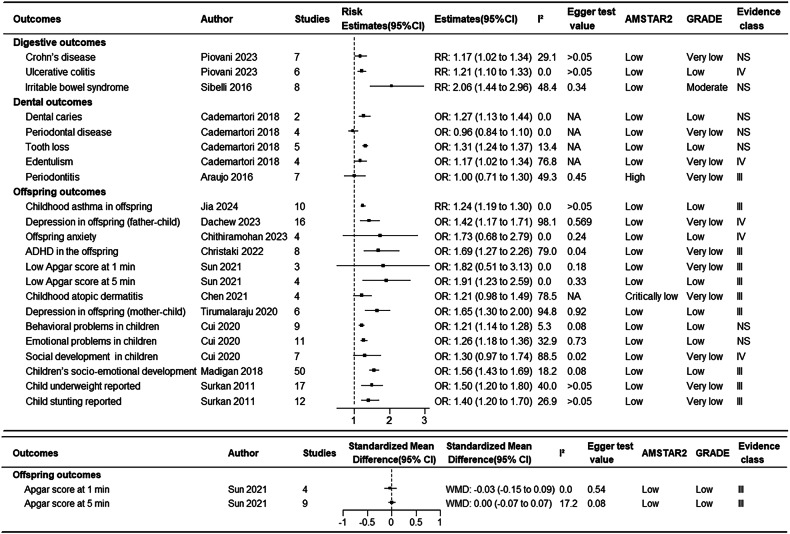
Associations between depression and digestive, dental and offspring health outcomes. NA not available, RR relative risk, OR odds ratio, HR hazard ratio, WMD weighted mean difference, AMSTAR2 A Measurement Tool to Assess Systematic Reviews 2, GRADE Grading of Recommendations, Assessment, Development, and Evaluation, ADHD attention deficit hyperactivity disorder.

**Fig. 5:**
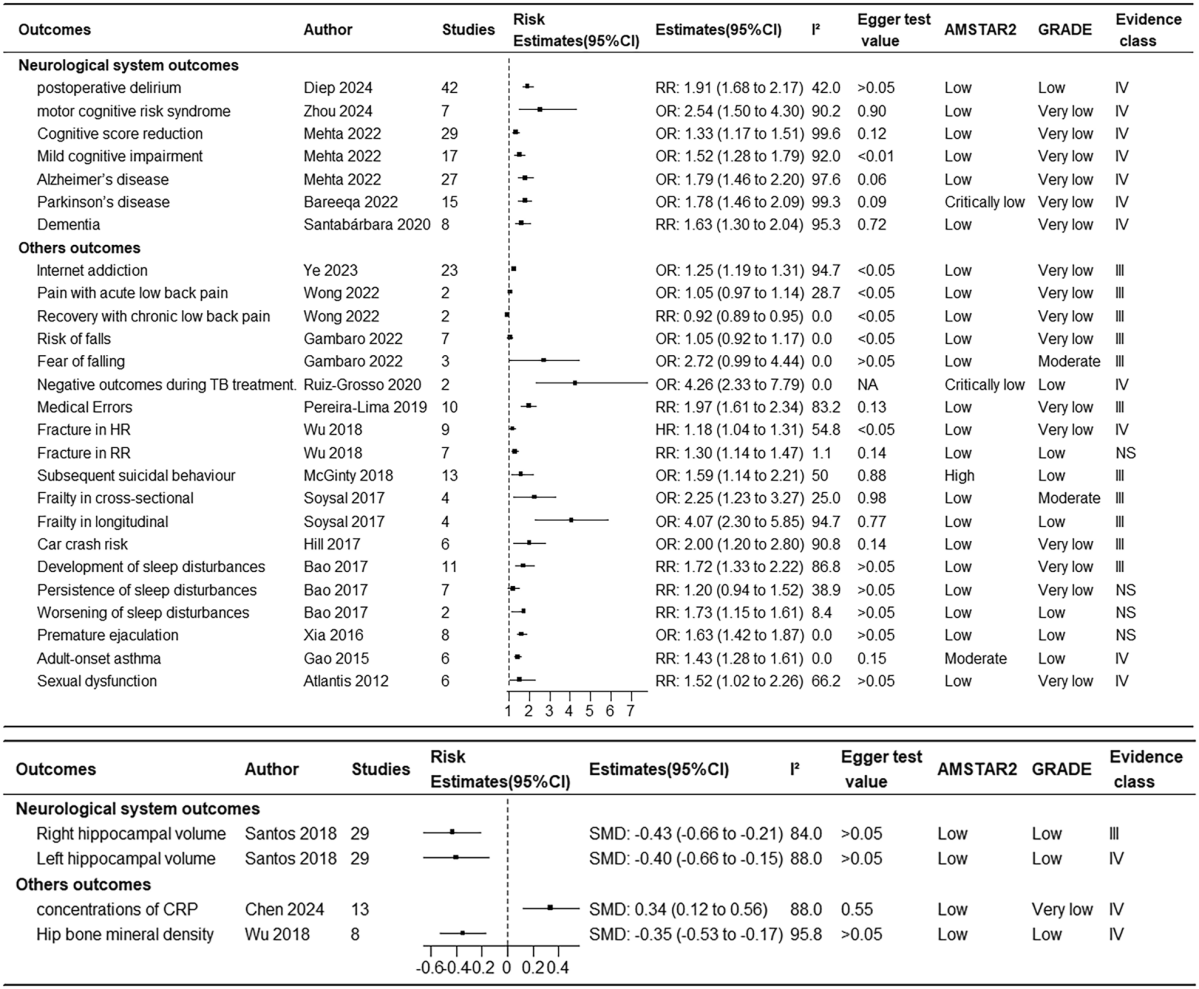
Associations between depression and neurological system and others health outcomes. NA not available, RR relative risk, OR odds ratio, HR hazard ratio, SMD standardized mean difference, AMSTAR2 A Measurement Tool to Assess Systematic Reviews 2, GRADE Grading of Recommendations, Assessment, Development, and Evaluation, CRP C-reactive protein, TB tuberculosis.

**Fig. 6:**
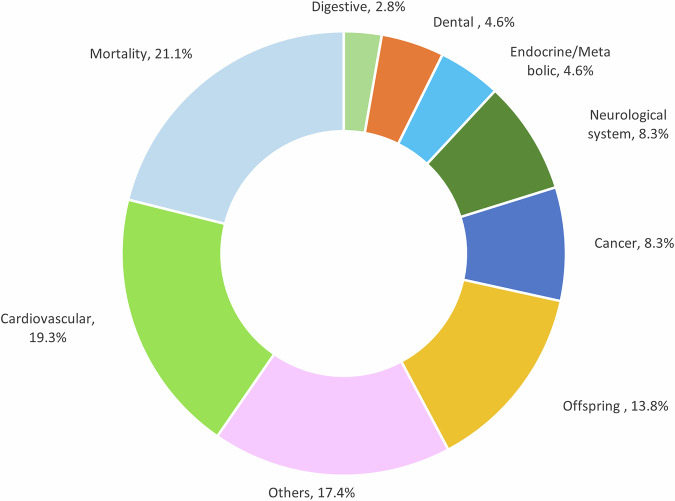
Map of health outcomes with depression.

Figure 1 shows the research selection process employed in this study. Following a systematic search, a total of 27,150 articles were retrieved from PubMed, Embase, and Web of Science, and the references of the included studies. After applying inclusion and exclusion criteria, 72 articles were found to meet the conditions, with some articles undergoing multiple meta-analyses, generating a total of 114 meta-analyses and 109 health outcomes. Table 1 summarizes the main characteristics of the conducted meta-analyses. Figure 2 shows the relationship between depression and outcomes related to cancer and mortality. Figure 3 illustrates the relationship between depression and endocrine/metabolic and cardiovascular outcomes. Figure 4 shows the relationship between depression and outcomes related to the digestive system, dental health, and offspring health. Figure 5 displays the relationship between depression and neurological outcomes as well as other types of related health outcomes. The distribution of health outcomes is presented in Fig. 6, with the majority of included meta-analyses focusing on the association between depression and mortality (*n* = 23, 21.1%). This was followed by cardiovascular outcomes (*n* = 21,19.8%), other health outcomes (*n* = 19, 17.4%), offspring health outcomes (*n* = 15, 13.8%), cancer (*n* = 9, 8.3%), neurological system health outcomes (*n* = 9, 8.3%), endocrine/metabolic health outcomes (*n* = 5, 4.6%), dental health outcomes (*n* = 5, 4.5%), and digestive system health outcomes (*n* = 3, 2.8%).

**Table 1:** Characteristics of the conducted meta-analyses^a^.

Outcomes	Author	Studies	Population	Sample size (cases/total)	Follow up	MA Metric	Estimates (95% CI)	T²
**Cancer outcomes**								
Cancer incidence	Wang 2020 [21]	21	general	1685782/NA	14.3 year (median)	RR	1.13 (1.06,1.19)	NA
Lung cancer incidence	Wang 2020 [21]	8	general	NA	NA	RR	1.41 (1.17,1.69)	NA
Oral cavity cancer incidence	Wang 2020 [21]	3	general	NA	NA	RR	1.47 (1.39,1.55)	NA
Prostate cancer incidence	Wang 2020 [21]	9	general	NA	NA	RR	1.37 (1.01,1.86)	NA
Skin cancer incidence	Wang 2020 [21]	3	general	NA	NA	RR	1.09 (1.01,1.18)	NA
Head and neck cancer overall survival	Van Der Elst 2021 [22]	7	head and neck cancer patient	NA/1743	NA	HR	1.33 (1.16,1.52)	0
Glioma survival outcome	Shi 2018 [23]	6	adult glioma patient	NA /6 to 87	ranging from 8 to 69.6 months	RR	0.51 (0.18 to 0.83)	NA
Breast cancer	Sun 2015 [24]	11	free of any subtypes of cancer at the beginning	2353/182,241	ranging from 5 to 38 years	RR	1.13(0.94,1.36)	NA
Breast cancer recurrence	Wang 2020 [21]	7	with breast cancer	NA/6716	8.2 years (median)	HR	1.24(1.07,1.43)	NA
**Mortality outcomes**								
All-cause mortality	Wei 2019 [12]	49	community-dwelling;≥60 years	27,910/190,152	ranging from 2 to 15 years	RR	1.34(1.27,1.42)	NA
CVD mortality	Wei 2019 [12]	15	community-dwelling;≥60 years	11,708/5244,426	ranging from 3.2 to 15 years	RR	1.31(1.20–1.43)	NA
Overall survival after transplantation in HSCT	Guillaume 2023 [31]	8	adults undergoing HSCT	NA/20,934	4.5 years (median)	HR	1.06(1.03–1.10)	<0.01
All cause mortality with PAD	Scierka 2023 [13]	4	≥18years of age with PAD	63,638/158,115	ranging from31.83 to 70.8 months	HR	1.24(1.07–1.45)	<0.01
All cause mortality in cancer	Wang 2020 [21]	20	general	NA	NA	RR	1.24 (1.13–1.35)	NA
All cause mortality in lung cancer	Wang 2020 [21]	5	general	NA	NA	RR	1.34 (1.16–1.55)	NA
All cause mortality in breast cancer	Wang 2020 [21]	12	with breast cancer,	NA/157,840	7.4 years (median)	HR	1.30(1.23–1.36)	NA
Specific mortality in bladder cancer	Wang 2020 [21]	2	general	NA	NA	RR	2.02 (1.29–3.17)	NA
Specific mortality in colorectum cancer	Wang 2020 [21]	2	general	NA	NA	RR	1.38 (1.23–1.55)	NA
Specific mortality in hematopoietic cancer	Wang 2020 [21]	2	general	NA	NA	RR	1.66 (1.43–1.93)	NA
Specific mortality in kidney cancer	Wang 2020 [21]	2	general	NA	NA	RR	1.85 (1.55–2.22)	NA
Specific mortality in prostate cancer	Wang 2020 [21]	3	general	NA	NA	RR	1.87 (1.62–2.16)	NA
All cause mortality in PCI	Song 2020 [15]	5	underwent PCI	NA/3405	5years (median)	RR	1.76(1.45–2.13)	<0.01
PSD mortality	Cai 2019 [16]	14	aged ≥18 years	141,487/250,071	ranging from 1 to 15 years.	HR	1.59(1.30–1.96)	0.10
All cause mortality in HF	Gathright 2017 [29]	14	≥ 18 years; diagnosed with HF	NA/6772	2.75 years (median)	HR	1.20(1.10–1.31)	NA
Coronary mortality	Wu 2016 [9]	8	depression is the predictor; CHD death is the outcome.	4292/170,271	8.45 years (median)	HR	1.36(1.14–1.63)	NA
All cause mortality in CABG	Stenman 2016 [30]	7	with HF	NA/89,490	ranging from 3 to 9.3 years	HR	1.41(1.19–1.63)	0.02
Organ post-transplant mortality	Dew 2016 [32]	20	solid organ transplant recipients	NA/51,921	5.8 years (median)	RR	1.42(0.98–1.86)	0.49
CKD Mortality	Palmer 2013 [27]	22	adults with CKD	NA/83,381	ranging from 3 months to 6.5 years	RR	1.47(1.25–1.68)	0.09
All cause mortality with diabetes by self-reports	Hofmann 2013 [26]	8	with diabetes	NA/12,809	ranging from 3 to 10 years	HR	2.51(1.85–3.17)	0.68
All cause mortality with diabetes by clinical interviews supported	Hofmann 2013 [26]	6	with diabetes	NA/87,560	ranging from 2 to 10 years	HR	1.34(1.04–1.63)	0.07
All cause mortality in MI	Meijer 2011 [28]	17	hospitalized for MI; depression was measured within 3 months after	892/10,362	16 months (median)	OR	2.25(1.73–2.93)	NA
Dying in the 2 years after the initial assessment with CHD	Barth 2004 [83]	7	with CHD or report data of a subgroup with CHD	NA/3228	ranging from 2 to 15 years	OR	2.24(1.39–3.60)	11.04
Dying in the long-term with CHD	Barth 2004 [83]	7	with CHD or report data of a subgroup with CHD	NA/3751	ranging from 4 months to 2 years	OR	1.78(1.12–2.83)	20.92
**Endocrine/Metabolic outcomes**								
Gestational diabetes mellitus	Zhang 2023 [36]	9	pregnant females	NA/127,195	NA	OR	1.19(1.02–1.36)	0.02
Diabetic nephropathy	Fang 2022 [35]	6	general	NA/945,683	NA	OR	1.22(1.13–1.31)	NA
Metabolic syndrome with cross-sectional studies	Moradi 2021 [37]	31	general	NA/111,866	NA	OR	1.48(1.33–1.64)	0.03
Metabolic syndrome with cohort studies	Moradi 2021 [37]	18	general	NA/287,628	NA	RR	1.38(1.17–1.64)	0.10
Type 2 diabetes	Graham 2020 [34]	15	general	NA/192,424	6 years (median)	RR	1.17(1.09–1.25)	0.00
Obesity	Mannan 2016 [33]	9	18 years and over;developed countries	NA/85,405	ranging from 5 to 22 years	RR	1.34(1.24–1.44)	<0.01
**Cardiovascular outcomes**								
Incident atrial fibrillation	Fu 2019 [38]	9	general	NA/1559,378	10 years (median)	RR	1.13(1.01–1.26)	0.02
Heart failure	Cao 2022 [7]	6	general without HF	4727/131,282	10 years (median)	HR	1.23(1.08–1.41)	NA
Coronary heart disease	Cao 2022 [8]	26	depression without CHD	NA/402,597	10 years (median)	RR	1.21(1.14–1.29)	NA
MACE outcomes with PAD	Abi-Jaoude 2022 [14]	3	with PAD	3820/117,062	NA	RR	0.96(0.53–1.39)	0.08
MALE outcomes with PAD	Abi-Jaoude 2022 [14]	4	with PAD	23,160/272,668	NA	RR	1.18(1.06–1.29)	<0.01
Risk of Readmission in HF	Kewcharoen 2021 [45]	10	diagnosed with HF	NA/53,165	NA	HR	1.45(1.17–1.79)	NA
MACEs after PCI	Song 2020 [15]	6	including patients that underwent PCI;	NA/2146	2.25 years (median)	RR	1.72(1.38–2.07)	<0.01
Non-fatal CVD events with type 2 diabetes	Inoue 2020 [42]	11	with diabetes	NA/1017628	6 years (median)	RR	1.35(1.20–1.53)	NA
Fatal CVD events with type 2 diabetes.	Inoue 2020 [42]	8	with diabetes	NA/20,930	6.5 years (median)	RR	1.47(1.21–1.77)	NA
A composite outcome following PCI	Zhang 2019 [44]	8	with CAD and receiving coronary stent implantation	NA/3297	3 years (median)	RR	1.42(1.23–1.61)	<0.01
Recurrent stroke event	Wu 2019 [46]	6	stroke patients	NA/4648	2.25 years (median)	RR	1.48(1.22–1.79)	NA
Diabetes complication: macrovascular and microvascular	Nouwen 2019 [43]	11	adults (>18 years old)	NA/2892,142	5.5 years (median)	HR	1.39(1.33–1.44)	<0.01
Ventricular arrhythmias	Fu 2019 [38]	9	NA	NA/3611	NA	HR	1.33(1.02–1.73)	NA
Coronary artery calcification	Lin 2018 [39]	12	participants were aged 35–84 years	NA/24,862	NA	OR	1.15(1.04–1.28)	NA
Sudden cardiac death	Shi 2017 [40]	4	general	NA/83,659	ranging from 0 to 10.4 years	HR	1.62(1.37–1.92)	NA
Ventricular tachycardia/ventricular fibrillation	Shi 2017 [40]	8	general	NA/4048	ranging from 0 to 10.4 years	HR	1.47(1.23–1.76)	NA
Myocardial Infarction	Wu 2016 [9]	9	general	4568/190,216	13 years (median)	HR	1.31(1.09–1.57)	NA
First-ever stroke	Barlinn 2015 [10]	28	nonhospitalized adults with no history of stroke or transient ischemic attack (TIA)	13,436/681,139	ranging from 3 to 29 years	HR	1.40(1.27–1.53)	NA
Hypertension	Meng 2012 [41]	9	general	NA/22,367	9.6 years (median)	RR	1.42(1.09–1.86)	0.28
Cardiac event in MI	Meijer 2011 [28]	18	general	2247/10,119	16 months (median)	OR	1.59(1.37–1.85)	NA
Cardiovascular diseases	Van der Kooy 2007 [47]	7	based on community-dwelling or general practice	NA/21,618	10.6 years (median)	OR	1.46(0.99–1.93)	0.21
**Digestive outcomes**								
Crohn’s disease	Piovani 2023 [48]	7	general	17,676/3386,186	ranging from 2.2 to 13 years.	RR	1.17(1.02–1.34)	0.01
Ulcerative colitis	Piovani 2023 [48]	6	general	28,165/3396,075	ranging from 2.2 to 13 years.	RR	1.21(1.10–1.33)	0.00
Irritable bowel syndrome	Sibelli 2016 [49]	8	with a GI infection aged 16 years	342/5007	from 3 months to 8 years	RR	2.06(1.44–2.96)	NA
**Neurological system outcomes**								
Postoperative delirium	Diep 2024 [66]	42	adults with pre-operative depression	NA/4664,051	NA	RR	1.91(1.68–2.17)	2.73
Motor cognitive risk syndrome	Zhou 2024 [63]	7	people over 60 years of age	NA/20,321	NA	OR	2.54(1.50–4.30)	NA
Cognitive score reduction	Mehta 2022 [62]	29	general	NA/279–7515	6.3 years (median)	OR	1.33(2.17–1.51)	0.10
Mild cognitive impairment	Mehta 2022 [62]	17	general	NA/181–6615	6.7 years (median)	OR	1.52(1.28–1.79)	0.08
Alzheimer’s disease	Mehta 2022 [62]	27	general	NA/185–2454,532	5.8 years (median)	OR	1.79(1.46–2.20)	0.21
Parkinson’s disease	Bareeqa 2022 [64]	15	40.8 to 71.45 years	256,801/1875,372	ranging from 3 to15 years	OR	1.78(1.46–1.09)	0.19
Dementia	Santabárbara 2020 [65]	8	over 50 years	NA/ 2476,454	6.8 years (median)	RR	1.63(1.30–2.04)	NA
Right hippocampal volume	Santos 2018 [67]	29	adults (≥18 years)	NA/2331	NA	SMD	−0.43(−0.66–−0.21)	0.31
Left hippocampal volume	Santos 2018 [67]	29	adults (≥18 years)	NA/2331	NA	SMD	−0.40(−0.66–−0.15)	0.43
**Offspring outcomes**								
Childhood asthma in offspring	Jia 2024 [57]	10	females and their children	NA/833,230	NA	RR	1.24(1.19–1.30)	0
Depression in offspring (father-child)	Dachew 2023 [52]	16	based on humans; father-child	NA/7153,723	NA	OR	1.42(1.17–1.71)	NA
Offspring anxiety	Chithiramohan 2023 [60]	4	adolescence and adulthood(≥12)	NA/1191	14.5 years (median)	OR	1.73(0.68–2.79)	<0.01
ADHD in offspring	Christaki 2022 [59]	8	general	NA/33,513	NA	OR	1.69(1.27–2.26)	NA
Apgar score at 1 min	Sun 2021 [55]	4	pregnant women	NA/1395	NA	MD	−0.03(−0.15–0.09)	NA
Low Apgar score at 1 min	Sun 2021 [55]	3	pregnant women	NA/1601	NA	OR	1.82(0.51–3.13)	NA
Apgar score at 5 min	Sun 2021 [55]	9	pregnant women	NA/2366	NA	MD	0.00(−0.07–0.07)	NA
Low Apgar score at 5 min	Sun 2021 [55]	4	pregnant women	NA/8608	NA	OR	1.91(1.23–2.59)	NA
Childhood atopic dermatitis	Chen 2021 [56]	4	children and their biological mothers	NA/110,064	NA	OR	1.21(0.98–1.49)	NA
Depression in offspring (mother-child)	Tirumalaraju 2020 [53]	6	mothers during their pregnancy and/or during the postnatal period;the offspring’s adolescence and adulthood	NA/19,535	NA	OR	1.65(1.30–2.00)	0.22
Behavioral problems in children	Cui 2020 [54]	9	paternal OR father OR men; offspring OR children OR child OR adolescent	NA/NA	17 or 18 weeks (median)	OR	1.21(1.14–1.28)	NA
Emotional problems in children	Cui 2020 [54]	11	paternal OR father OR men; offspring OR children OR child OR adolescent	NA/NA	18 weeks (median)	OR	1.26(1.18–1.36)	NA
Social development in children	Cui 2020 [54]	7	paternal OR father OR men; offspring OR children OR child OR adolescent	NA/NA	17 or 18 weeks (median)	OR	1.30(0.97–1.74)	NA
Children’s socio-emotional development	Madigan 2018 [58]	50	maternal with depression in pregnancy; offspring outcomes were collected prior to the age of 18 y	NA/33,211	NA	OR	1.56(1.43–1.69)	0.03
Child underweight reported	Surkan 2011 [61]	17	developing countries but applied no other population	NA/13,923	NA	OR	1.50(1.20–1.80)	NA
Child stunting reported	Surkan 2011 [61]	12	developing countries but applied no other population	NA/13,214	NA	OR	1.40(1.20–1.70)	NA
**Dental outcomes**								
Dental caries	Cademartori 2018 [50]	2	≥ 30 years	NA/4857	NA	OR	1.27(1.13–1.44)	NA
Periodontal disease	Cademartori 2018 [50]	4	≥ 30 years	NA/13,492	NA	OR	0.96(0.84–1.10)	NA
Tooth loss	Cademartori 2018 [50]	5	≥ 30 years	NA/5507,766	NA	OR	1.31(1.24–1.37)	NA
Edentulism	Cademartori 2018 [50]	4	≥ 30 years	NA/5592,964	NA	OR	1.17(1.02–1.34)	NA
Periodontitis	Araujo 2016 [51]	7	general	NA/6125	NA	OR	1.00(0.71–1.30)	0.07
**Others outcomes**								
Concentrations of CRP	Chen 2024 [70]	13	patients were clinically diagnosed as PSD	1004/3294	NA	SMD	0.34 (0.12–0.56)	0.14
Internet addiction	Ye 2023 [68]	23	primary, secondary and college students aged 10–24	NA/34,554	NA	OR	1.25(1.19–1.31)	NA
Pain with acute low back pain	Wong 2022 [80]	2	≥16 years with LBP/radiculopathy	NA/314	6 months (median)	OR	1.05(0.97–1.14)	<0.01
Recovery with chronic low back pain	Wong 2022 [80]	2	≥16 years with LBP/radiculopathy	NA/13,263	8.5 months (median)	RR	0.92(0.89–0.95)	<0.01
Risk of falls	Gambaro 2022 [81]	7	≥ 60 years	NA/32,368	NA	OR	1.05(0.92–1.17)	<0.01
Fear of falling	Gambaro 2022 [81]	3	≥ 60 years	NA/7339	NA	OR	2.72(0.99–4.44)	<0.01
Negative outcomes during TB treatment.	Ruiz-Grosso 2020 [71]	2	general	NA/973	NA	OR	4.26(2.33–7.79)	0.00
Medical Errors	Pereira-Lima 2019 [77]	10	practicing/resident physicians	NA/21,517	NA	RR	1.97(1.61–2.34)	0.26
Fracture with HR	Wu 2018 [72]	9	general	NA/309,862	7 years (median)	HR	1.18(1.04–1.31)	0.01
Fracture with RR	Wu 2018 [72]	7	general	NA/64,975	7 years (median)	RR	1.30(1.14–1.47)	<0.01
Hip bone mineral density	Wu 2018 [72]	8	general	NA/15,442	3.57 years (median)	SMD	−0.35(−0.53–−0.17)	0.05
Subsequent suicidal behavior	McGinty 2018 [79]	13	experiencing FEP	428/3002	24 months (median)	OR	1.22(0.84–1.59)	0.09
Frailty with cross-sectional	Soysal 2017 [74]	4	older adults≥ 60 years	867/2167	NA	OR	2.25(1.23–3.27)	0.28
Frailty with longitudinal	Soysal 2017 [74]	4	older adults≥ 60 years	NA/48,014	3 years (median)	OR	4.07(2.30–5.85)	2.93
Car crash risk	Hill 2017 [78]	6	NA	NA/349,435	NA	OR	2.00(1.20–2.80)	0.76
Development of sleep disturbances	Bao 2017 [69]	11	community-dwelling older adults (≥50 years; mean age ≥ 60 years),without sleep disturbances	NA/24,564	36 months (median)	RR	1.72(1.33–2.22)	NA
Persistence of sleep disturbances	Bao 2017 [69]	7	community-dwelling older adults (≥50 years; mean age ≥ 60 years),without sleep disturbances	NA/17,369	30 months (median)	RR	1.20(0.94–1.52)	NA
Worsening of sleep disturbances	Bao 2017 [69]	2	community-dwelling older adults (≥50 years; mean age ≥ 60 years),without sleep disturbances	NA/1744	30 months (median)	RR	1.73(1.15–1.61)	NA
Premature ejaculation	Xia 2016 [75]	8	>18 years suffering from PE	NA/18,053	NA	OR	1.63(1.42–1.87)	NA
Adult-onset asthma	Gao 2015 [73]	6	general	2334/83,684	ranging from 8 to 20 years	RR	1.43(1.28–1.61)	0.00
Sexual dysfunction	Atlantis 2012 [76]	6	adult populations	NA/3285	ranging from 2–9 years	RR	1.52(1.02–2.26)	NA

^a^*MA* meta-analyses, *NA* not available, *RR* relative risk, *OR* odds ratio, *HR* hazard ratio, *SMD* standardized mean difference, *WMD* weighted mean difference, *PAD* peripheral artery disease, *PCI* percutaneous coronary intervention, *PSD* post-stroke depression, *HF* heart failure, *CHD* coronary heart disease, *CABG* coronary artery bypass grafting, *CKD* chronic kidney disease, *MI* myocardial infarction, *HSCT* hematopoietic stem cell transplantation, *MACE* major adverse cardiovascular events, *MALE* major adverse limb events, *CVD* cardiovascular diseases, *CAD* coronary artery disease, *TIA* transient ischemic attack, *GI* gastrointestinal, *POD* postoperative delirium, *CABG* coronary artery bypass grafting, *CRP* C-reactive protein, *ADHD* attention deficit hyperactivity disorder, *TB* tuberculosis, *FEP* first episode psychosis, *PE* premature ejaculation.

### Cancer outcomes

A meta-analysis comprising 21 studies indicated that depression increased the incidence of cancer (RR = 1.13). In addition, the same study also found that depression increases the incidence of lung cancer (RR = 1.41), oral cancer (RR = 1.47), prostate cancer (RR = 1.37), and skin cancer (RR = 1.09) [21]. Patients with depression before the treatment of head and neck cancer have a worse overall survival rate compared to those without depression (HR = 1.33) [22]. Similarly, glioma patients with depression have poorer survival outcomes than those without depression (RR = 0.51, 95% CI 0.18–0.83) [23]. An analysis of 11 cohort studies concluded that current epidemiological evidence did not support a connection between depression and breast cancer (RR = 1.13, 95% CI 0.94–1.36) [24]. However, another meta-analysis found a positive correlation between depression and increased risk of breast cancer recurrence (HR = 1.24, 95% CI 1.07–1.43) [25] (Fig. 2).

### Mortality

In the community, late-life depression significantly increased all-cause mortality (RR = 1.34) and cardiovascular (CVD) mortality (RR = 1.33) [12] among the elderly. Assessments of depression based on self-reports (HR = 2.51) and clinical interviews (HR = 1.34) show a positive correlation between depression and the risk of all-cause mortality in diabetic patients [26]. Furthermore, depression elevated the risk of death for individuals with coronary artery disease (HR = 1.36) [9] and chronic kidney disease (CKD) (RR = 1.47) [27]. Depression not only increased the all-cause mortality among cancer patients (RR = 1.24) [21] but also heightened the all-cause mortality rates by 34 and 30% in lung cancer and breast cancer patients with depression, respectively. Furthermore, patients with bladder cancer, colorectal cancer, hematological cancers, kidney cancer, and prostate cancer who also have depression experience increases in cancer-specific mortality by 102, 38, 66, 85, and 87%, respectively [21]. Patients with peripheral artery disease (PAD) (HR = 1.06), stroke (HR = 1.59) [16], myocardial infarction (MI) (OR = 2.25) [28], and heart failure (HF) (HR = 1.20) [29] who had depression showed an increased all-cause mortality. Among patients with coronary heart disease (CHD), depression significantly raises mortality risk, with an OR of 2.24 for short-term (within 2 years) mortality and 1.78 for long-term mortality. Compared to patients without depression, those undergoing coronary artery bypass grafting (CABG) [30] or percutaneous coronary intervention (PCI) [15] with depression experienced a 41 and 76% increase in all-cause mortality. Depression had a significant impact on the overall survival of patients undergoing hematopoietic stem cell transplantation (HSCT), with a hazard ratio of 1.06 (HR = 1.06) [31]. However, another meta-analysis of 20 cohort studies found no association between depression and post-transplant mortality (RR = 1.42, 95% CI 0.98–1.86) [32]. (Fig. 2).

### Endocrine/metabolic outcomes

A meta-analysis of 9 cohort studies showed individuals with depression had a 34% higher risk of obesity than those without [33]. Furthermore, depression significantly increased the risk of developing diabetes (RR = 1.17) [34], diabetic nephropathy (OR = 1.22) [35], and gestational diabetes (OR = 1.19) [36]. Base on evidence from 31 cross-sectional studies and 18 cohort studies, patients with depression had a higher likelihood of suffering from metabolic syndrome compared to those without depression, with odds ratios of 1.48 and risk ratios of 1.38 [37] (Fig. 3).

### Cardiovascular outcomes

Compared to non-depressed individuals, those with depressive symptoms were likelier to face various cardiovascular problems, including atrial fibrillation (RR = 1.13) [6], heart failure (HR = 1.23), coronary heart disease (RR = 1.21) [7], ventricular arrhythmias (HR = 1.33) [38], coronary artery calcification (OR = 1.15) [39], sudden cardiac death (HR = 1.62) [40], ventricular tachycardia/ventricular fibrillation (HR = 1.47) [40], myocardial infarction (HR = 1.31) [9], first stroke (HR = 1.40) [10], hypertension (RR = 1.42) [41], cardiac events following myocardial infarction (OR = 1.59) [28], and major adverse limb events in PAD (RR = 1.18) [14]. In the diabetic population, depressive symptoms significantly increased the risk of non-fatal and fatal cardiovascular events, with risk ratios of 1.35 and 1.47 [42], and diabetes complications involving both large and small blood vessels (HR = 1.39) [43]. PAD patients with depression undergoing percutaneous coronary intervention faced higher risks of adverse outcomes (RR = 1.42) [44], including major adverse cardiovascular events (RR = 1.72) [15]. Furthermore, heart failure patients with depression had increased risks of readmission [45] and stroke recurrence [46] by 45 and 48%. However, some studies indicated no significant link between depression and cardiovascular disease (OR = 1.46, 95% CI 0.99–1.93) [47] or major cardiovascular adverse events in PAD (RR = 0.96, 95% CI 0.53–1.39) [14] (Fig. 3).

### Digestive and dental health outcomes

Figure 4 shows the meta-analyses on the association between depression and digestive and dental health outcomes. Depression increased the risk of Crohn’s disease (RR = 1.17) and ulcerative colitis (RR = 1.21) [48], and individuals with depression were twice as likely to suffer from irritable bowel syndrome [49] as those without depression. Moreover, depression seemed to increase the risk of oral diseases in adults and the elderly, especially cavities (OR = 1.27), tooth loss (OR = 1.31) [50], and edentulism (OR = 1.17, 95% CI 1.02–1.34) [50]. However, the association between depression and periodontitis (OR = 0.96, 95% CI 0.84–1.10) [50] as well as gingivitis (OR = 1.00, 95% CI 0.71–1.30) [51] was not significant.

### Offspring health outcomes

Paternal depression significantly increases the risk of depression in offspring (OR = 1.42) [52], while maternal perinatal depression has an even greater impact on the risk of depression in offspring (OR = 1.70) [53]. Similarly, paternal perinatal depression was associated with an increase in behavioral and emotional issues in offspring by 21 and 26%, respectively [54]. Maternal depression during pregnancy raised the risk of low Apgar scores in newborns (OR = 1.91) [55] and was linked to asthma (OR = 1.24) [56, 57] as well as social and emotional challenges (OR = 1.56) [58] in children. Offspring of mothers with postpartum depression faced an elevated risk of attention deficit hyperactivity disorder (OR = 1.69) [59], though the impact on anxiety disorders was not significant (OR = 1.73, 95% CI 0.68–2.79) [60]. Additionally, children of mothers with depression or depressive symptoms were more prone to being underweight (OR = 1.50) [61] and experiencing developmental delays (OR = 1.40) [61]. However, studies have shown that prenatal depression was not associated with atopic dermatitis in children [56] or low 1-min [55] and 5-min Apgar scores [55] in newborns. Paternal perinatal depression was related to a statistically non-significant increase in children’s social functioning (OR = 1.30, 95% CI 0.97–1.74) [54] (Fig. 4).

### Neurological system health outcomes

Compared to healthy individuals, patients with depression experienced a reduction in subsequent cognitive scores, an increased risk of mild cognitive impairment, Alzheimer’s disease, Parkinson’s disease, dementia, and motor cognitive risk syndrome, with respective increases of 33, 52, 79, 78, 63, and154% [62–65]. Patients with depression had a 91% increased risk of developing postoperative delirium after surgery [66] Additionally, depression linked to atrophy in both right and left hippocampal regions (SMD = −0.43, 95% CI −0.66–−0.21) [67] and (SMD = −0.40, 95% CI −0.66–−0.15) [67] (Fig. 5).

### Others health outcomes

Depression had a broad and profound impact on various health outcomes across different age groups and professions. In adolescents, depression significantly increased the risk of internet addiction (OR = 1.25, 95% CI 1.19–1.31) [68]. Among the elderly, those with depression were more likely to experience sleep disturbances (RR = 1.72) and worsening symptoms (RR = 1.73) [69]. Additionally, depressive symptoms were linked to worsening recovery in patients with chronic lumbar disc herniation (RR = 0.92, 95% CI 0.89–0.95), adverse outcomes in tuberculosis treatment (OR = 4.26, 95% CI 2.33–7.79), reduced hip bone mineral density (SMD = −0.35, 95% CI −0.53–−0.17), increased incidence of adult-onset asthma (RR = 1.43, 95% CI 1.28–1.61), and elevated C-reactive protein (CRP) concentrations in patients with PSD (SMD = 0.34, 95% CI 0.12–0.56) [70–73]. Studies reporting risk ratios and hazard ratios found that the risk of fractures in patients with depression increases by 18 and 30%, respectively [72]. Patients with depression were more likely to be frail (OR = 2.25; OR = 4.07), premature ejaculation (OR = 1.63), and sexual dysfunction (RR = 1.52) [74–76]. Doctors who exhibit depressive symptoms are at a higher risk of committing medical errors (RR = 1.97) [77]. Depression was also related to a higher risk of car accidents (OR = 2.00) [78]. A meta-analysis of 13 cohort studies showed that patients with depression symptoms were 59% more likely to engage in suicidal behavior during follow-up than non-depressed individuals [79]. However, symptoms of depression did not correlate with pain intensity in acute lumbar disc herniation patients (OR = 1.05, 95% CI 0.97–1.14) [80]. Elderly patients with depression were not significantly linked to falls (OR = 1.05, 95% CI 0.92–21.17), fear of falling (OR = 2.72, 95% CI 0.99–4.44) [81], or persistent sleep disorders (RR = 1.20, 95% CI 0.94–1.52) [69] (Fig. 5).

### Heterogeneity

Reanalysis found that approximately 58 (50.9%) out of the 114 studies that we reanalyzed had significant heterogeneity (I^2^ > 50% or *P* value of Cochran’s Q test <0.1). Of these, 42 (36.8%) meta-analyses showed high heterogeneity (I^2^ > 75%).

### Assessment of risk of bias

In our reanalysis, significant publication bias was detected in studies concerning all-cause mortality, cardiovascular mortality, overall survival in hematopoietic stem cell transplantation, all-cause mortality in cancer, heart failure, coronary artery mortality, post-transplant mortality, metabolic syndrome in cross-sectional and cohort studies, coronary heart disease, myocardial infarction, offspring attention deficit hyperactivity disorder, offspring social function, mild cognitive impairment, internet addiction, pain intensity in acute lumbar disc herniation, exacerbation of chronic lumbar disc herniation, falls and fractures (Egger test *P* value < 0.05).The remaining studies did not exhibit significant publication bias or could not be assessed for publication bias due to an insufficient number of studies.

### Amstar, grade, and evidence classification

The AMSTAR 2 analysis revealed that the methodological quality of 7 studies (6.1%) on all-cause mortality in diabetics with depression (self-reported and clinical interviews), all-cause mortality in PAD, coronary heart disease, hypertension periodontitis, and suicidal behavior were classified as “High” quality. Additionally, 4 studies (3.5%) concerning mortality in CKD, major cardiovascular adverse events in PAD, major adverse limb events in PAD, and adult-onset asthma were rated as “Moderate” quality, while 95 studies (83.3%) received a “Low” or “Critically Low” quality (Table S2).

According to GRADE scoring, 7 studies (6.1%) covering bladder cancer mortality, all-cause mortality post-myocardial infarction, mortality risk within two years of coronary heart disease, major adverse cardiovascular events post-percutaneous coronary intervention, irritable bowel syndrome, fear of falling, and frailty were deemed to have “Moderate” epidemiological evidence quality. The epidemiological evidence quality of 107 studies (93.9%) were considered “Low” or “Very Low” (Table S3).

Regarding the classification of evidence, 55 out of 114 outcomes (48.2%) were rated as “III” (suggestive evidence), 40 (35.1%) as “ IV” (weak evidence), and 19 (16.7%) were deemed “NS” (non-significant) (Table S4).

## Discussion

### Principal findings and possible explanations

Our review encompassed 72 articles, including 114 meta-analyses and 109 health outcomes, and showed depression’s link to adverse health effects. Depression was found to be associated with all-cause mortality and various disease-specific mortality, such as cardiovascular death, coronary artery disease, and lung cancer mortality. Depression was also linked to multiple adverse cardiovascular outcomes, including atrial fibrillation, coronary heart disease, and stroke. Furthermore, depression increased the risk of developing cancers, such as lung cancer, oral cancer, and prostate cancer. Depression also raised the risk of neurological disorders, including Alzheimer’s disease, Parkinson’s disease, and dementia. Our study further indicated that depression was associated with endocrine-metabolic diseases (such as diabetes, obesity, and diabetic nephropathy), digestive system diseases (such as Crohn’s disease and ulcerative colitis), oral diseases (such as dental caries and tooth loss), offspring health outcomes (such as offspring depression and low infant Apgar scores), sleep disorders, adult asthma, traffic accidents, and premature ejaculation, among other adverse health outcomes.

The AMSTAR2 tool assessed articles’ methodological quality on depression’s impact on health outcomes, and the GRADE method analyzed evidence quality. For the methodological quality, only 11 meta-analyses (all-cause mortality in diabetic patients (self-reported and clinical interviews), all-cause mortality in PAD, coronary heart disease, hypertension, mortality in CKD patients, major cardiovascular adverse events in PAD patients, major adverse limb events in PAD patients, periodontitis, suicidal behavior, and adult-onset asthma were rated as “high” or “moderate” in methodological quality. The other health outcomes were found to have “Low” or “Critically low” methodological quality, mainly due to a lack of consideration for publication bias or because the authors did not provide a detailed list of excluded studies with justification for the exclusions. The failure to assess the quality of the original studies also reduced the overall quality of the research [7, 42, 43, 47, 56, 71, 82]. Moreover, the evidence quality for studies on cancer, digestive system, oral health, offspring health, and endocrine and metabolic outcomes was generally not high, with only 7 outcomes (bladder cancer mortality, all-cause mortality after myocardial infarction, mortality risk within two years for coronary heart disease, major adverse cardiovascular events after percutaneous coronary intervention, irritable bowel syndrome, fear of falling, and frailty) having a “moderate” level of evidence quality. The primary reasons for the low quality of evidence were high heterogeneity among studies [6, 8, 9, 12, 14, 16, 21, 22, 24, 26, 27, 29, 37, 39, 42, 43, 54, 59, 61–63, 67–70, 72, 76, 78, 52], lack of precision [6, 14, 21, 23, 24, 31, 38, 39, 48, 50, 55, 63, 69, 70, 76, 80, 82], or wide confidence intervals [21, 28, 45, 49, 63, 74, 81–83]. Therefore, future meta-analyses related to depression and health outcomes could reduce publication bias by systematically searching various databases, including unpublished studies, and using comprehensive search strategies to capture all relevant research. It is recommended that authors provide a detailed list of excluded studies with clear reasons for the exclusions to ensure transparency and replicability of the process. Improving study quality also hinges on strictly adhering to research design principles, including, but not limited to, proper randomization methods, allocation concealment, blinding, and appropriate statistical analysis techniques. Researchers should also ensure sufficient sample sizes to achieve statistical significance, thereby enhancing the study’s power.

Depression had linked to an increased all-cause and cardiovascular mortality, particularly pronounced among the elderly. This was consistent with findings from two large cohort studies, the China Kadoorie Biobank study and the Dongfeng-Tongji study, which had shown elevated rates of all-cause mortality (HR = 1.21, HR = 1.45) and cardiovascular mortality (HR = 1.33) in depressed patients aged 65 and over [84]. Elderly vulnerability may arise from social isolation, bereavement, health deterioration, and cognitive decline. Early detection and effective management of depression in this demographic were deemed crucial for mitigating its potential adverse effects on health and lifespan. The review also uncovered that in diabetic, depression, whether assessed through self-reported or clinically interviewed, could increase risk of all-cause mortality [26]. The association between self-reported depression and all-cause mortality was notably stronger than that measured through clinical interviews, possibly reflecting the sensitivity of self-reporting in capturing the subjective experience of depressive symptoms. This underscored the importance of considering the method of depression measurement in future studies. It is noteworthy that depression not only increased the mortality rates among specific disease groups, such as patients with coronary artery disease, but also adversely affected the mortality rates of patients undergoing certain surgical treatments, such as those who had undergone coronary artery bypass graft surgery or had received hematopoietic stem cell transplants [30, 31]. A multi-center prospective study had aligned with our findings, showing a positive correlation between depressive symptoms within a year after coronary artery bypass graft surgery and mortality [85]. This could be indirectly attributed to depressed patients struggling to adhere to recommendations for a healthy diet, regular exercise, and medication due to low mood, lack of energy, and reduced motivation. Although our analysis had revealed an association between depression and increased mortality risk, no significant connection was observed in terms of mortality post-organ transplantation and PAD mortality [14, 32]. This suggested a degree of heterogeneity in the impact of depression on different mortality, warranting further investigation in future research. AMSTAR2 and GRADE analyses had indicated that the quality of research for 7 disease outcomes was “High” or “Moderate”. However, the quality of evidence for most studies linking depression to mortality remained low. Therefore, future studies would need to employ more rigorous research designs and methodologies to enhance the quality of evidence.

A retrospective cohort study spanning 5.5 years revealed that over one-tenth of patients with depression developed cardiovascular diseases, with a particularly notable finding that individuals with prolonged durations of depression faced a higher risk of cardiovascular diseases after adjustments were made for multiple variables [86]. This discovery aligned with our comprehensive review, which noted that individuals exhibiting symptoms of depression were more likely to encounter a range of cardiovascular issues, including atrial fibrillation, heart failure, coronary artery disease, myocardial infarction, hypertension [6–10, 41], and significant limb problems due to PAD [14]. Patients with depression often endured sustained psychological stress, leading to chronic stress responses that activated the HPA axis and the autonomic nervous system, causing increases in blood pressure and heart rate, thereby elevating the risk of cardiovascular diseases [87]. The emergence or worsening of cardiovascular diseases could, in turn, exacerbate symptoms of depression, creating a vicious cycle. For instance, cardiovascular diseases could restrict physical activity, further intensifying symptoms of depression [88]. However, a reevaluation of a meta-analysis encompassing seven cohort studies found no significant association between depression and cardiovascular diseases [47], which could be attributed to differences in study designs, methods of assessing depression, sample characteristics, or statistical approaches. Future research should employ standardized tools for assessing depression, control for potential confounding factors, and conduct long-term follow-ups to accurately assess the relationship between depression and cardiovascular diseases.

Our umbrella review revealed that depression, as a risk factor, increased the incidence of cancer, particularly lung cancer, oral cancer, prostate cancer, and skin cancer [21]. Depression was also linked to poorer survival outcomes in patients with head and neck cancers and gliomas [22, 23], suggesting that depression could not only elevate the likelihood of developing cancer but might also adversely affect the treatment responses and quality of life of cancer patients. This could be attributed to factors such as the unhealthy lifestyles of individuals with depression such as smoking and excessive alcohol consumption, diminished immune function due to chronic stress, or reduced adherence to treatment [87, 89, 90]. However, the epidemiological evidence connecting depression with breast cancer outcomes appeared insufficient. Although some meta-analyses identified a positive correlation between depression and the risk of recurrence in breast cancer [25], the high heterogeneity and imprecision of these studies rendered the quality of evidence low, necessitating a cautious interpretation of this conclusion.

Exposure to depression was found to have increased the risk of obesity, diabetes and its complications, as well as metabolic syndrome [33–36]. Studies indicated that the risk of obesity in patients with depression had risen by 34%, which could be attributed to their reduced physical activity, unhealthy dietary habits, and metabolic changes associated with chronic stress related to depression [87]. Additionally, chronic stress and depression could promote obesity by affecting hormone levels, such as an increase in cortisol. Metabolic syndrome, a key risk factor for cardiovascular diseases and diabetes, was diagnosed in 27.7% of patients with depression in a study from northwestern India [91]. This study also highlighted that individuals with metabolic syndrome engaged in less physical activity and had poorer dietary habits [92, 93] compared to those without metabolic syndrome, suggesting that depression might indirectly heighten the risk of metabolic syndrome by impacting lifestyle factors. Our analysis also revealed limitations in current research, with all included studies rated as “low” in methodological quality, and the quality of evidence for most endocrine/metabolic outcomes being “Low” or “Very low.” This underscores the necessity for further large-scale prospective studies.

As a common mental disorder, depression was found to have complex associations with neurological conditions such as mild cognitive impairment, Alzheimer’s disease, motor cognitive risk syndrome, Parkinson’s disease, dementia, and hippocampal atrophy [62, 64, 67]. For instance, a study conducted on hospitalized adolescents revealed that clinical depression correlated with cognitive features [94], suggesting that depression might have begun impacting the brain early on and potentially accelerated cognitive decline and the progression of neurodegenerative diseases. Moreover, the link between depression and physical changes in the brain was also confirmed to some extent. Research showed that depression could lead to a more rapid decrease in hippocampal volume, which is closely linked to impairments in memory and other cognitive functions, thereby potentially speeding up the development of diseases like Alzheimer’s disease [95]. However, it is crucial to acknowledge the limitations present in studies exploring the relationship between depression and neurological diseases, such as small sample sizes, insufficient control of confounding factors, and heterogeneity in diagnostic criteria [82]. Although evidence indicated a connection between the depression and neurological diseases, the methodological and evidence quality of these studies was generally low due to publication bias and high heterogeneity, limiting our understanding of the depth and mechanisms of these relationships.

Additionally, depression was found to have a positive correlation with various digestive system diseases and oral health issues. Specifically, depression increased the risk of Crohn’s disease, ulcerative colitis, and irritable bowel syndrome [48, 49]. Depression also impacted the oral health of adults and the elderly, such as cavities, tooth loss, and edentulism [50]. A longitudinal study of elderly individuals in the UK further reinforced these findings, showing that participants who exhibited symptoms of depression at baseline were more likely to report poorer self-assessed oral health [96]. This suggests that depression might indirectly affect dental health by impacting individuals’ quality of life, including emotional state, physical vitality, social interactions, and hygiene habits. The use of antidepressant medications could exacerbate this situation [97], as they may cause dry mouth, cariogenic dietary habits, and decreased immune function following oral infections, thereby increasing the risk of cavities or other dental issues. The influence of depression on various digestive system diseases was also supported by a large-scale Mendelian randomization study. This study indicated that depression increased the risk of developing 12 types of digestive system diseases, including irritable bowel syndrome, non-alcoholic fatty liver disease, alcoholic liver disease, and gastroesophageal reflux disease [98]. Additionally, experiments inducing depressive-like behavior in mice found that the depressive state might increase the susceptibility to intestinal inflammation by affecting the function of the vagus nerve [99], further supporting the biological link between depression and digestive system diseases.

Parents exposed to depression increased the risk of negative health outcomes for their offspring, including mental health issues such as depression, anxiety, and attention deficit hyperactivity disorder [59, 60], as well as physical health problems like asthma, low Apgar scores, child underweight, and child developmental delays [55, 61]. The stability of the family environment, emotional support, parenting styles, and parents’ interaction methods profoundly influenced children’s mental health and social adaptability. Compared to mothers, fathers’ depression had a lesser impact on children’s social functions, which is related to mothers often being the primary caregivers and emotional supporters in many cultures and family structures [100]. Additionally, pregnancy is a critical period for child development, and the psychological and physical health of the mother can affect fetal development through various mechanisms, such as reduced blood flow to the fetus [101], increased cortisol levels potentially entering the offspring’s growth environment through the placenta, or increased maternal inflammatory cytokines and serotonin [102]. In a study using a mouse model to investigate the effects of prenatal maternal depression on offspring, socially isolated mothers in a depressed state led their offspring to exhibit increased anxiety-like behaviors, cognitive performance changes, and alterations in the amygdala transcriptome in adulthood [103]. Therefore, prevention and intervention for depressed parents and their offspring’s health, such as early screening, psychosocial support, and designing intervention measures, are particularly important.

In our study, we identified significant associations between depression and multiple unique health outcomes, including not only psychological and behavioral issues such as internet addiction and sleep disorders [68, 69] but also severe physical health consequences like suicide, exacerbation of chronic pain, osteoporosis, asthma, and increased risks of fractures and frailty [73, 74, 79]. The link between depression and sleep disorders was especially noteworthy, as sleep problems [69] are not only common symptoms of depression but can also exacerbate the severity of depression, creating a vicious cycle. Moreover, the increase in medical accidents among doctors highlighted the potential impact of depression on professional performance and occupational safety [77], which needs attention in the health management of medical professionals. Depression could affect individuals’ attention and response capabilities, leading to an increased risk of car accidents, potentially exacerbated by the use of antidepressants in depressed patients [78].

Depression not only affected mental health but also extensively impacted individuals’ physical health, daily functioning, and social interactions as well as offspring related health outcomes. Although more high-quality evidence is still needed on the health effects of depression, the findings of the present umbrella review provide more evidence that the health effects of depression are serious, longstanding, and affecting majority of health system as well as the offspring health, which stress the importance of early screening, timely interventions, and regular monitoring of depression in public health and clinical practice.

### Strengths and limitations

Our comprehensive review systematically summarizes previous meta-analyses on the relationship between depression and health outcomes, generating 109 health outcomes. These include cardiovascular outcomes, mortality, cancer, offspring health outcomes, neurological outcomes, endocrine/metabolic health outcomes, dental and gastrointestinal health outcomes, and offspring related outcomes. Our umbrella review assessed the impact of depression on various health outcomes, encompassing a wide range of potential health issues, from cardiovascular health to mortality and mental health, allowing for a more comprehensive evaluation of overall patient risk and a more holistic approach to addressing their health concerns, rather than solely focusing on the occurrence of depression. A systematic and comprehensive search strategy was employed from three scientific databases: PubMed, Embase, and Web of Science. Where possible, standardized methods were used to replicate each meta-analysis, including employing random-effects analyses, and generating measures for heterogeneity and publication bias for better comparison of outcomes. Besides providing an extensive overview of the available evidence, we also critically evaluate the methodological quality of the meta-analyses and the quality of evidence for all the reported associations. Three standard methods were used including AMSTAR2, GRADE, and the evidence classification standard. The methodological quality of the included meta-analyses was assessed using the AMSTAR2 tool [17], followed by the evaluation of the evidence quality for each outcome using the GRADE tool [18]. Outcomes were categorized into four classes based on the evidence classification standard [19] to assess our confidence in the estimates and to enhance decision-making quality in clinical practice.

Although Köhler’s umbrella review focuses on depression as a risk factor for various outcomes (such as genetics, environmental exposures) [104], this review is the first to consider depression as the exposure and various health outcomes as the study outcomes. Additionally, existing reviews are mostly limited to singular outcomes like mortality [11], while this paper fills the gap in systematic evidence regarding health outcomes with depression as the exposure, emphasizing the long-term impact of depression on multiple diseases and its public health significance.

The present study has several limitations. First, randomized controlled trials are better suited to identify causal effects compared with cohort studies. However, trials of the long-term effects of depression on the risk of hard end points (such as CVD, cancer, and dementia) are lacking, being unfeasible to conduct because of their high cost and lack of adherence to long-term interventions. No evidence was rated as high-quality for observational studies in this umbrella review, which may have led to some bias in the interpretation of the findings. To increase the quality of evidence, more experimental studies comparing the interventions on depression with health-related outcomes should be conducted and included in systematic reviews. Secondly, 65.8% of the association analyses (*n* = 75) were conducted with fewer than 10 primary studies; thus, the interpretation of these outcomes might be limited due to the small number of studies. In addition, selection bias may have affected the representativeness of the included studies, as we included only published literature and excluded grey literature and unpublished studies, which may have led to over-estimation or under-estimation of some findings. Recall bias is also a potential limitation of this study, especially in some retrospective studies, where participants’ memories of depression and health outcomes may not be completely accurate, thus affecting the reliability of the data. Finally, to achieve more rigorous and reproducible umbrella review research, it is necessary to adopt standardized methods to reduce the overlap of reviews caused by subjective decisions and different methodologies [105]. We made decisions to select the eligible reviews based on previous umbrella reviews published in leading biomedical journals [19, 106–108]. Nonetheless, future umbrella reviews should compare how different methods of selection and analyses influence the results of the umbrella review, thereby improving the quality and consistency of the research.

## Conclusions

To sum up, depression was adversely related to a multitude of health outcomes. In our umbrella review, the quality of epidemiological evidence was considered moderate for bladder cancer mortality, all-cause mortality in myocardial infarction, mortality within two years for patients with coronary heart disease, major adverse cardiovascular events after percutaneous coronary intervention, irritable bowel syndrome, fear of falling, and frailty. Evidence for other health outcomes remains limited. Due to the scarcity of high-quality evidence, further large-scale, multicenter, and international randomized controlled trials or prospective studies are needed to validate the impact of depression on various human health outcomes and arrive at more definitive conclusions.

## Supplementary information

Supplementary Material

PRISMA_2020_checklis
