# Supplementary material for: Depression and health outcomes: An umbrella review of systematic reviews and meta-analyses of observational studies
Source: Transl Psychiatry. 2025 Aug 20;15:298. doi: 10.1038/s41398-025-03463-8 (PMC12368087; doi:10.1038/s41398-025-03463-8)
Supplement: Supplementary file 1 — Supplementary Material [file 41398_2025_3463_MOESM1_ESM.docx]

**Depression and Health Outcomes: An Umbrella Review of Systematic Reviews and Meta-analyses of Observational Studies**

**Authors:**

Xiaojuan Chen ^1^, Xiaoning Liu ^2^, Fengjuan Li ^2^, Haitian He ^3^, Xinying Li ^2^, Tianhang Qin ^4^, Bin Jiang ^5^, Yuge Chen ^6^, Yanqi Wang ^2^, Yuhao Su ^2^, Xiaojie Wang ^5^, Lei Liang ^6^, Huiling Hua ^6^, Jun Wu ^5^, Jianping Ma ^5^, Fulan Hu ^7^, Pei Qin ^8*^

**Institutional address:**

^1^School of Public Health, Shantou University, Shantou, Guangdong, China

^2^Department of Respiratory and Critical Care Medicine, the Second Affiliated Hospital of Zhengzhou University, Zhengzhou, China

^3^Urology Department of Shenzhen Qianhai Shekou Free Trade Zone Hospital

^4^Institute of Software Chinese Academy of Sciences, Beijing, Guangdong, China

^5^Department of Neurology, Shenzhen Qianhai Shekou Free Trade Zone Hospital, Shenzhen, Guangdong, China

^6^Department of Gynecology and Obstetrics, Shenzhen Qianhai Shekou Free Trade Zone Hospital, Shenzhen, Guangdong, China

^7^Department of Biostatistics and Epidemiology, School of Public Health, Shenzhen University Medical School, Shenzhen, China

^8^Department of Behavioural Science and Health, University College London, London, UK

***Corresponding author**

Dr. Pei Qin, M.D., Ph.D.

**Institute:** Department of Behavioural Science and Health, University College

London

**Address:** 1-19 Torrington Place, London WC1E 7HB, UK

**E-mail:** **p.qin@ucl.ac.uk**

**Table S1.** Umbrella Review: search terms and strategy used

**Table S2.** AMSTAR 2 results for individual studies included in the umbrella review

**Table S3.** GRADE classification of quality of evidence for depression and health outcomes

**Table S4.** Evidence classification for individual studies included in the umbrella review

**Table S1.** Umbrella Review: search terms and strategy used

| **Search terms for** **PubMed (n=5,638), until December 17, 2024** |
| --- |
| #1 (depression[title/abstract]) OR (depressive[title/abstract]) OR (depression[Mesh]) OR (major depressive disorder[Mesh])  583,628results |
| #2 "systematic review" OR "systematic literature review" OR "meta-analysis" OR "meta-analyses" OR "meta analysis" OR "meta analyses"  548,231 results |
| #3 "prospective studies"[Mesh] OR "cohort studies"[Mesh] OR "longitudinal studies"[Mesh] OR "prospective"[Title/Abstract] OR "longitudinal"[Title/Abstract] OR "cohort"[Title/Abstract] OR "cohorts"[Title/Abstract] OR "follow-up"[Title/Abstract] OR “followed up"[Title/Abstract]  4,176,538 results |
| #1 AND #2 AND #3  5,638 results |
| **Search terms for Embase (n=11,985), until December 17, 2024** |
| #1'depression'/exp OR 'depression' OR ' depressive ' OR ' major depressive disorder '  1,041,044 4results |
| #2'systematic review' OR 'systematic literature review' OR 'meta-analysis' OR 'meta-analyses' OR 'meta analysis' OR 'meta analyses'  833,169 results |
| #3 'cohort analysis' OR 'longitudinal study' OR 'prospective study' OR 'follow up' OR 'prospective' OR 'longitudinal' OR 'cohort' OR 'cohorts' OR 'follow-up' OR 'followed up'  5,512,734 results |
| #1 AND #2 AND #3  11,985 results |
| **Search terms for Web of Science (n=9,527), until December 17, 2024** |
| #1 TS=(Depression OR depression OR depressive OR major depressive disorder)  1,030,338 results |
| #2 TS=(systematic review OR systematic literature review OR meta-analysis OR meta-analyses OR meta analysis OR meta analyses)  837,716 results |
| #3 TS=(cohort analysis OR longitudinal study OR prospective study OR follow up OR prospective OR longitudinal OR cohort OR cohorts OR follow-up OR followed up )  5,046,410 results |
| #1 AND #2 AND #3  9,527 results |

**Table S2.** AMSTAR 2 results for individual studies included in the umbrella review^1^

| **Outcomes** | **Study** | **AMSTAR 2 Items ^a,b^** | | | | | | | | | | | | | | | | **Quality** |
| --- | --- | --- | --- | --- | --- | --- | --- | --- | --- | --- | --- | --- | --- | --- | --- | --- | --- | --- |
|  |  | 1^a^ | 2^b^ | 3^a^ | 4^b^ | 5^a^ | 6^a^ | 7^b^ | 8^a^ | 9^b^ | 10^a^ | 11^b^ | 12^a^ | 13^b^ | 14^a^ | 15^b^ | 16^a^ |  |
| **Cancer outcomes** |  |  |  |  |  |  |  |  |  |  |  |  |  |  |  |  |  |  |
| Cancer incidence | Wang 2020 | Y | Y | Y | Y | Y | Y | N | Y | Y | Y | Y | Y | Y | Y | Y | Y | **Low** |
| Lung cancer incidence | Wang 2020 | Y | Y | Y | Y | Y | Y | N | Y | Y | Y | Y | Y | Y | Y | Y | Y | **Low** |
| Oral cavity cancer incidence | Wang 2020 | Y | Y | Y | Y | Y | Y | N | Y | Y | Y | Y | Y | Y | Y | Y | Y | **Low** |
| Prostate cancer incidence | Wang 2020 | Y | Y | Y | Y | Y | Y | N | Y | Y | Y | Y | Y | Y | Y | Y | Y | **Low** |
| Skin cancer incidence | Wang 2020 | Y | Y | Y | Y | Y | Y | N | Y | Y | Y | Y | Y | Y | Y | Y | Y | **Low** |
| Head and neck cancer overall survival | Van Der Elst 2021 | Y | PY | Y | Y | Y | Y | N | Y | Y | Y | Y | Y | Y | Y | Y | Y | **Low** |
| Glioma survival outcome | Shi 2018 | Y | Y | Y | Y | Y | Y | N | Y | Y | Y | Y | Y | Y | Y | Y | N | **Low** |
| Breast cancer | Sun 2015 | Y | PY | Y | Y | Y | Y | N | Y | Y | Y | Y | Y | Y | Y | Y | N | **Low** |
| Breast cancer recurrence | Xuan Wang 2020 | Y | PY | Y | Y | Y | Y | N | Y | Y | Y | Y | Y | Y | Y | Y | Y | **Low** |
| **Mortality outcomes** |  |  |  |  |  |  |  |  |  |  |  |  |  |  |  |  |  |  |
| All-cause mortality | Wei 2019 | Y | PY | Y | Y | Y | Y | N | Y | Y | N | Y | Y | Y | Y | Y | Y | **Low** |
| CVD mortality | Wei 2019 | Y | PY | Y | Y | Y | Y | N | Y | Y | N | Y | Y | Y | Y | Y | Y | **Low** |
| Overall survival after transplantation in HSCT | Guillaume 2023 | Y | Y | Y | PY | Y | Y | N | Y | Y | N | Y | Y | Y | Y | Y | Y | **Low** |
| All cause mortality with PAD | Scierka 2023 | Y | PY | Y | Y | Y | Y | Y | Y | Y | Y | Y | Y | Y | Y | Y | Y | **High** |
| All cause mortality in cancer | Wang 2020 | Y | Y | Y | Y | Y | Y | N | Y | Y | Y | Y | Y | Y | Y | Y | Y | **Low** |
| All cause mortality in lung cancer | Wang 2020 | Y | Y | Y | Y | Y | Y | N | Y | Y | Y | Y | Y | Y | Y | Y | Y | **Low** |
| All cause mortality in breast cancer | Xuan Wang 2020 | Y | PY | Y | Y | Y | Y | N | Y | Y | Y | Y | Y | Y | Y | Y | Y | **Low** |
| Specific mortality in bladder cancer | Wang 2020 | Y | Y | Y | Y | Y | Y | N | Y | Y | Y | Y | Y | Y | Y | Y | Y | **Low** |
| Specific mortality in colorectum cancer | Wang 2020 | Y | Y | Y | Y | Y | Y | N | Y | Y | Y | Y | Y | Y | Y | Y | Y | **Low** |
| Specific mortality in hematopoietic cancer | Wang 2020 | Y | Y | Y | Y | Y | Y | N | Y | Y | Y | Y | Y | Y | Y | Y | Y | **Low** |
| Specific mortality in kidney cancer | Wang 2020 | Y | Y | Y | Y | Y | Y | N | Y | Y | Y | Y | Y | Y | Y | Y | Y | **Low** |
| Specific mortality in prostate cancer | Wang 2020 | Y | Y | Y | Y | Y | Y | N | Y | Y | Y | Y | Y | Y | Y | Y | Y | **Low** |
| All cause mortality in PCI | Song 2020 | Y | PY | Y | PY | Y | Y | N | Y | Y | Y | Y | Y | Y | Y | Y | Y | **Low** |
| PSD mortality | Cai 2019 | Y | PY | Y | PY | Y | Y | N | Y | Y | Y | Y | Y | Y | Y | Y | Y | **Low** |
| All cause mortality in HF | Gathright 2017 | Y | PY | Y | PY | Y | Y | N | Y | Y | Y | Y | Y | Y | Y | Y | Y | **Low** |
| Coronary mortality | Wu 2016 | Y | PY | Y | PY | Y | Y | N | Y | Y | Y | Y | Y | Y | Y | Y | Y | **Low** |
| All cause mortality in CABG | Stenman 2016 | Y | PY | Y | Y | Y | Y | N | Y | Y | Y | Y | Y | Y | Y | Y | Y | **Low** |
| Organ post-transplant mortality | Dew 2016 | Y | PY | Y | Y | Y | Y | N | Y | Y | Y | Y | Y | Y | Y | Y | Y | **Low** |
| CKD Mortality | Palmer 2013 | Y | PY | Y | Y | Y | Y | Y | Y | Y | Y | Y | Y | Y | Y | Y | N | **Moderate** |
| All cause mortality with diabetes by self-reports | Hofmann 2013 | Y | PY | Y | Y | Y | Y | Y | Y | Y | Y | Y | Y | Y | Y | Y | Y | **High** |
| All cause mortality with diabetes by clinical interviews supported | Hofmann 2013 | Y | PY | Y | Y | Y | Y | Y | Y | Y | Y | Y | Y | Y | Y | Y | Y | **High** |
| All cause mortality in MI | Meijer 2011 | Y | PY | Y | Y | Y | Y | N | Y | Y | N | Y | Y | Y | Y | Y | N | **Low** |
| Dying in the 2 years after the initial assessment with CHD | Barth 2004 | Y | PY | Y | Y | Y | Y | N | Y | Y | N | Y | Y | Y | Y | Y | Y | **Low** |
| Dying in the long-term with CHD | Barth 2004 | Y | PY | Y | Y | Y | Y | N | Y | Y | N | Y | Y | Y | Y | Y | Y | **Low** |
| **Endocrine/Metabolic outcomes** |  |  |  |  |  |  |  |  |  |  |  |  |  |  |  |  |  |  |
| Gestational diabetes mellitus | Zhang 2023 | Y | PY | Y | PY | Y | Y | N | Y | Y | N | Y | Y | Y | Y | Y | N | **Low** |
| Diabetic nephropathy | Fang 2022 | Y | Y | Y | Y | Y | Y | N | Y | N | Y | Y | N | Y | Y | Y | Y | **Low** |
| Metabolic syndrome with cross-sectional studies | Moradi 2021 | Y | PY | Y | Y | Y | Y | N | Y | Y | Y | Y | Y | Y | Y | Y | Y | **Low** |
| Metabolic syndrome with cohort studies | Moradi 2021 | Y | PY | Y | Y | Y | Y | N | Y | Y | Y | Y | Y | Y | Y | Y | Y | **Low** |
| Type 2 diabetes | Graham 20220 | Y | PY | Y | Y | Y | Y | N | Y | Y | Y | Y | Y | Y | Y | Y | Y | **Low** |
| Obesity | Mannan 2016 | Y | PY | Y | Y | Y | Y | N | Y | Y | Y | Y | Y | Y | Y | Y | Y | **Low** |
| **Cardiovascular outcomes** |  |  |  |  |  |  |  |  |  |  |  |  |  |  |  |  |  |  |
| Incident atrial fibrillation | Fu 2022 | Y | PY | Y | PY | Y | Y | N | Y | Y | Y | Y | Y | Y | Y | Y | Y | **Low** |
| Heart failure | Lihuan Cao 2022 | Y | PY | Y | Y | Y | Y | N | Y | Y | Y | Y | N | Y | Y | N | Y | **Critically low** |
| Coronary heart disease | Hongfu Cao 2022 | Y | PY | Y | Y | Y | Y | Y | Y | Y | Y | Y | N | Y | Y | Y | Y | **High** |
| MACE outcomes with PAD | Abi-Jaoude 2022 | Y | PY | Y | PY | Y | Y | Y | Y | Y | Y | Y | Y | Y | Y | Y | Y | **Moderate** |
| MALE outcomes with PAD | Abi-Jaoude 2022 | Y | PY | Y | PY | Y | Y | Y | Y | Y | Y | Y | Y | Y | Y | Y | Y | **Moderate** |
| Risk of Readmission in HF | Kewcharoen 2021 | Y | PY | Y | PY | Y | Y | N | Y | Y | Y | Y | Y | Y | Y | Y | Y | **Low** |
| MACEs after PCI | Song 2020 | Y | PY | Y | PY | Y | Y | N | Y | Y | Y | Y | Y | Y | Y | Y | Y | **Low** |
| Non-fatal CVD events with type 2 diabetes | Inoue 2020 | Y | PY | Y | PY | Y | Y | N | Y | N | Y | Y | Y | Y | Y | Y | Y | **Critically low** |
| Fatal CVD events with type 2 diabetes. | Inoue 2020 | Y | PY | Y | PY | Y | Y | N | Y | N | Y | Y | Y | Y | Y | Y | Y | **Critically low** |
| A composite outcome following PCI | Zhang 2019 | Y | PY | Y | PY | Y | Y | N | Y | Y | Y | Y | Y | Y | Y | Y | Y | **Low** |
| Recurrent stroke event | Wu 2019 | Y | PY | Y | Y | Y | Y | N | Y | Y | N | Y | Y | Y | Y | Y | Y | **Low** |
| Diabetes complication: macrovascular and microvascular | Nouwen 2019 | Y | PY | Y | PY | Y | Y | N | Y | Y | Y | Y | Y | Y | Y | N | Y | **Critically low** |
| Ventricular arrhythmias | Fu 2019 | Y | PY | Y | PY | Y | Y | N | Y | Y | Y | Y | Y | Y | Y | Y | Y | **Low** |
| Coronary artery calcification | Lin 2018 | Y | PY | Y | Y | Y | Y | N | Y | Y | Y | Y | Y | Y | Y | Y | N | **Low** |
| Sudden cardiac death | Shi 2017 | Y | PY | Y | PY | Y | Y | N | Y | Y | Y | Y | Y | Y | Y | Y | Y | **Low** |
| Ventricular tachycardia/ventricular fibrillation | Shi 2017 | Y | PY | Y | PY | Y | Y | N | Y | Y | Y | Y | Y | Y | Y | Y | Y | **Low** |
| Myocardial Infarction | Wu 2016 | Y | PY | Y | PY | Y | Y | N | Y | Y | Y | Y | Y | Y | Y | Y | Y | **Low** |
| First-ever stroke | Barlinn 2015 | Y | PY | Y | Y | Y | Y | N | Y | Y | Y | Y | Y | Y | Y | Y | Y | **Low** |
| Hypertension | Meng 2012 | Y | PY | Y | Y | Y | Y | Y | Y | Y | Y | Y | Y | Y | Y | Y | Y | **High** |
| Cardiac event in MI | Meijer 2011 | Y | PY | Y | Y | Y | Y | N | Y | Y | N | Y | Y | Y | Y | Y | N | **Low** |
| Cardiovascular diseases | Van der Kooy 2007 | Y | PY | Y | PY | Y | Y | N | Y | Y | Y | Y | Y | Y | Y | N | N | **Critically low** |
| **Digestive outcomes** |  |  |  |  |  |  |  |  |  |  |  |  |  |  |  |  |  |  |
| Crohn’s disease | Piovani 2023 | Y | PY | Y | Y | Y | Y | N | Y | Y | Y | Y | Y | Y | Y | Y | Y | **Low** |
| Ulcerative colitis | Piovani 2023 | Y | PY | Y | Y | Y | Y | N | Y | Y | Y | Y | Y | Y | Y | Y | Y | **Low** |
| Irritable bowel syndrome | Sibelli 2016 | Y | PY | Y | Y | Y | Y | N | Y | Y | Y | Y | Y | Y | Y | Y | Y | **Low** |
| **Neurological system outcomes** |  |  |  |  |  |  |  |  |  |  |  |  |  |  |  |  |  |  |
| Postoperative delirium | Diep 2024 | Y | PY | Y | Y | Y | Y | N | Y | Y | Y | Y | Y | Y | Y | Y | Y | **Low** |
| Motor cognitive risk syndrome | Zhou 2024 | Y | PY | Y | Y | Y | Y | N | Y | Y | Y | Y | Y | Y | Y | Y | Y | **Low** |
| Cognitive score reduction | Mehta 2022 | Y | Y | Y | Y | Y | Y | N | Y | Y | Y | Y | Y | Y | Y | Y | Y | **Low** |
| Mild cognitive impairment | Mehta 2022 | Y | Y | Y | Y | Y | Y | N | Y | Y | Y | Y | Y | Y | Y | Y | Y | **Low** |
| Alzheimer’s disease | Mehta 2022 | Y | Y | Y | Y | Y | Y | N | Y | Y | Y | Y | Y | Y | Y | Y | Y | **Low** |
| Parkinson’s disease | Bareeqa 2022 | Y | PY | Y | Y | Y | Y | N | Y | Y | N | Y | Y | Y | Y | N | N | **Critically low** |
| Dementia | Santabárbara 2020 | Y | PY | Y | Y | Y | Y | N | Y | Y | N | Y | Y | Y | Y | Y | N | **Low** |
| Right hippocampal volume | Santos 2018 | Y | PY | Y | Y | Y | Y | N | Y | N | N | Y | Y | Y | Y | Y | Y | **Low** |
| Left hippocampal volume | Santos 2018 | Y | PY | Y | Y | Y | Y | N | Y | N | N | Y | Y | Y | Y | Y | Y | **Low** |
| **Offspring outcomes** |  |  |  |  |  |  |  |  |  |  |  |  |  |  |  |  |  |  |
| Childhood asthma in offspring | Jia 2024 | Y | PY | Y | Y | Y | Y | N | Y | Y | Y | Y | Y | Y | Y | Y | Y | **Low** |
| Depression in offspring (father-child) | Dachew 2023 | Y | PY | Y | Y | Y | Y | N | Y | Y | Y | Y | Y | Y | Y | Y | Y | **Low** |
| Offspring anxiety | Chithiramohan 2023 | Y | PY | N | Y | N | N | N | Y | Y | Y | Y | N | Y | Y | Y | Y | **Low** |
| ADHD in offspring | Christaki 2022 | Y | PY | Y | Y | Y | Y | N | Y | Y | Y | Y | Y | Y | Y | Y | Y | **Low** |
| Apgar score at 1 min | Sun 2021 | Y | PY | Y | Y | Y | Y | N | Y | Y | Y | Y | Y | Y | N | Y | Y | **Low** |
| Low Apgar score at 1 min | Sun 2021 | Y | PY | Y | Y | Y | Y | N | Y | Y | Y | Y | Y | Y | N | Y | Y | **Low** |
| Apgar score at 5 min | Sun 2021 | Y | PY | Y | Y | Y | Y | N | Y | Y | Y | Y | Y | Y | N | Y | Y | **Low** |
| Low Apgar score at 5 min | Sun 2021 | Y | PY | Y | Y | Y | Y | N | Y | Y | Y | Y | Y | Y | N | Y | Y | **Low** |
| Childhood atopic dermatitis | Chen 2021 | Y | PY | N | Y | Y | Y | N | Y | N | Y | Y | Y | Y | Y | N | Y | **Critically low** |
| Depression in offspring (mother-child) | Tirumalaraju 2020 | Y | PY | Y | PY | Y | Y | N | Y | Y | Y | Y | Y | Y | Y | Y | Y | **Low** |
| Behavioral problems in children | Cui 2020 | Y | PY | Y | Y | Y | Y | N | Y | Y | N | Y | Y | Y | Y | Y | Y | **Low** |
| Emotional problems in children | Cui 2020 | Y | PY | Y | Y | Y | Y | N | Y | Y | N | Y | Y | Y | Y | Y | Y | **Low** |
| Social development in children | Cui 2020 | Y | PY | Y | Y | Y | Y | N | Y | Y | N | Y | Y | Y | Y | Y | Y | **Low** |
| Children’s socio-emotional development | Madigan 2018 | Y | PY | Y | Y | Y | Y | N | Y | Y | N | Y | Y | Y | Y | Y | Y | **Low** |
| Child underweight reported | Surkan 2011 | Y | Y | Y | Y | Y | Y | N | Y | Y | Y | Y | Y | Y | Y | Y | Y | **Low** |
| Child stunting reported | Surkan 2011 | Y | Y | Y | Y | Y | Y | N | Y | Y | Y | Y | Y | Y | Y | Y | Y | **Low** |
| **Dental outcomes** |  |  |  |  |  |  |  |  |  |  |  |  |  |  |  |  |  |  |
| Dental caries | Cademartori 2018 | Y | PY | Y | PY | Y | Y | N | Y | Y | Y | Y | Y | Y | Y | Y | Y | **Low** |
| Periodontal disease | Cademartori 2018 | Y | PY | Y | PY | Y | Y | N | Y | Y | Y | Y | Y | Y | Y | Y | Y | **Low** |
| Tooth loss | Cademartori 2018 | Y | PY | Y | PY | Y | Y | N | Y | Y | Y | Y | Y | Y | Y | Y | Y | **Low** |
| Edentulism | Cademartori 2018 | Y | PY | Y | PY | Y | Y | N | Y | Y | Y | Y | Y | Y | Y | Y | Y | **Low** |
| Periodontitis | Araujo 2016 | Y | Y | Y | Y | Y | Y | Y | Y | Y | Y | Y | Y | Y | Y | Y | Y | **High** |
| **Others outcomes** |  |  |  |  |  |  |  |  |  |  |  |  |  |  |  |  |  |  |
| Concentrations of CRP | Chen 2024 | Y | PY | Y | Y | Y | Y | N | Y | Y | Y | Y | Y | Y | Y | Y | Y | **Low** |
| Internet addiction | Ye 2023 | Y | PY | Y | Y | Y | Y | N | PY | Y | Y | Y | Y | Y | Y | Y | Y | **Low** |
| Pain with acute low back pain | Wong 2022 | Y | Y | Y | Y | Y | Y | N | Y | Y | Y | Y | Y | Y | Y | Y | Y | **Low** |
| Recovery with chronic low back pain | Wong 2022 | Y | Y | Y | Y | Y | Y | N | Y | Y | Y | Y | Y | Y | Y | Y | Y | **Low** |
| Risk of falls | Gambaro 2022 | Y | Y | Y | PY | Y | Y | N | Y | Y | Y | Y | Y | Y | Y | Y | Y | **Low** |
| Fear of falling | Gambaro 2022 | Y | Y | Y | PY | Y | Y | N | Y | Y | Y | Y | Y | Y | Y | Y | Y | **Low** |
| Negative outcomes during  TB treatment. | Ruiz-Grosso 2020 | Y | PY | Y | PY | Y | Y | N | Y | Y | Y | Y | Y | Y | Y | N | Y | **Critically low** |
| Medical Errors | Pereira-Lima 2019 | Y | PY | Y | PY | Y | Y | N | Y | Y | Y | Y | Y | Y | Y | Y | Y | **Low** |
| Fracture with HR | Wu 2018 | Y | PY | Y | Y | Y | Y | N | Y | Y | Y | Y | Y | Y | Y | Y | Y | **Low** |
| Fracture with RR | Wu 2018 | Y | PY | Y | Y | Y | Y | N | Y | Y | Y | Y | Y | Y | Y | Y | Y | **Low** |
| Hip bone mineral density | Wu 2018 | Y | PY | Y | Y | Y | Y | N | Y | Y | Y | Y | Y | Y | Y | Y | Y | **Low** |
| Subsequent suicidal behavior | McGinty 2018 | Y | PY | Y | Y | Y | Y | Y | Y | Y | Y | Y | Y | Y | Y | Y | Y | **High** |
| Frailty with cross-sectional | Soysal 2017 | Y | PY | Y | Y | Y | Y | N | Y | Y | N | Y | Y | Y | Y | Y | Y | **Low** |
| Frailty with longitudinal | Soysal 2017 | Y | PY | Y | Y | Y | Y | N | Y | Y | N | Y | Y | Y | Y | Y | Y | **Low** |
| Car crash risk | Hill 2017 | N | PY | Y | PY | Y | Y | N | Y | Y | Y | Y | Y | Y | Y | Y | Y | **Low** |
| Development of sleep disturbances | Bao 2017 | Y | PY | Y | PY | Y | Y | N | Y | Y | Y | Y | Y | Y | Y | Y | Y | **Low** |
| Persistence of sleep disturbances | Bao 2017 | Y | PY | Y | PY | Y | Y | N | Y | Y | Y | Y | Y | Y | Y | Y | Y | **Low** |
| Worsening of sleep disturbances | Bao 2017 | Y | PY | Y | PY | Y | Y | N | Y | Y | Y | Y | Y | Y | Y | Y | Y | **Low** |
| Premature ejaculation | Xia 2016 | Y | Y | Y | Y | Y | Y | N | Y | Y | Y | Y | Y | Y | Y | Y | Y | **Low** |
| Adult-onset asthma | Gao 2015 | Y | PY | Y | Y | Y | Y | Y | Y | Y | Y | Y | Y | Y | Y | Y | N | **Moderate** |
| Sexual dysfunction | Atlantis 2012 | Y | PY | Y | Y | Y | Y | N | Y | Y | N | Y | Y | Y | Y | Y | Y | **Low** |

^a^The following 16 items are covered in AMSTAR 2:

1.Did the research questions and inclusion criteria for the review include the components of PICO?

2.Did the report of the review contain an explicit statement that the review methods were established prior to the conduct of the review and did the report justify any significant deviations from the protocol?

3.Did the review authors explain their selection of the study designs for inclusion in the review?

4.Did the review authors use a comprehensive literature search strategy?

5.Did the review authors perform study selection in duplicate?

6.Did the review authors perform data extraction in duplicate?

7.Did the review authors provide a list of excluded studies and justify the exclusions?"

8.Did the review authors describe the included studies in adequate detail?"

9.Did the review authors use a satisfactory technique for assessing the risk of bias (RoB) in individual studies that were included in the review?

10.Did the review authors report on the sources of funding for the studies included in the review?

11.If meta-analysis was performed, did the review authors use appropriate methods for statistical combination of results?"

12.If meta-analysis was performed, did the review authors assess the potential impact of RoB in individual studies on the results of the meta-analysis or other evidence synthesis? "

13.Did the review authors account for RoB in primary studies when interpreting/discussing the results of the review?"

14.Did the review authors provide a satisfactory explanation for, and discussion of, any heterogeneity observed in the results of the review?"

15.If they performed quantitative synthesis, did the review authors carry out an adequate investigation of publication bias (small study bias) and discuss its likely impact on the results of the review?"

16.Did the review authors report any potential sources of conflict of interest, including any funding they received for conducting the review?"

^b^Items 2, 4, 7, 9, 11, 13, 15 were critical domains.

**High:** No or one non-critical weakness: the systematic review provides an accurate and comprehensive summary of the results of the available studies that address the question of interest

**Moderate:** More than one non-critical weakness: the systematic review has more than one weakness but no critical flaws. It may provide an accurate summary of the results of the available studies that were included in the review

**Low:** One critical flaw with or without non-critical weaknesses: the review has a critical flaw and may not provide an accurate and comprehensive summary of the available studies that address the question of interest

**Critically** **low**：More than one critical flaw with or without non-critical weaknesses: the review has more than one critical flaw and should not be relied on to provide an accurate and comprehensive summary of the available studies.

^1^MA: meta-analyses; NA: not available; RR: relative risk; OR: odds ratio; HR: hazard ratio; SMD : standardized mean difference; WMD: weighted mean difference ;PAD :peripheral artery disease; PCI: percutaneous coronary intervention ;PSD: post-stroke depression; HF: heart failure; CHD: coronary heart disease ;CABG: coronary artery bypass grafting; CKD: chronic kidney disease; MI: myocardial infarction; HSCT: hematopoietic stem cell transplantation ; MACE: major adverse cardiovascular events; MALE: major adverse limb events; CVD: cardiovascular diseases; CAD: coronary artery disease; TIA : transient ischemic attack; GI: gastrointestinal; POD :postoperative delirium ;CABG: coronary artery bypass grafting; ADHD: attention deficit hyperactivity disorder; CRP: C-reactive protein ; TB: tuberculosis; FEP: first episode psychosis; PE: premature ejaculation

**Table S3.** GRADE classification of quality of evidence for depression and health outcomes^1^

| **Outcome** | **Study** | **Study design** | **Risk of Bias** | **Inconsistency** | **Indirectness** | **Imprecision** | **Publication bias** | **Magnitude  of effect** | **Plausible Confounding** | **Dose-response gradient** | **Quality** |
| --- | --- | --- | --- | --- | --- | --- | --- | --- | --- | --- | --- |
| **Cancer outcomes** |  |  |  |  |  |  |  |  |  |  |  |
| Cancer incidence | Wang 2020 | 21cohort | Not serious | serious | Not serious | Not serious | undetected | no | would not reduce effect | no | **Very low** |
| Lung cancer incidence | Wang 2020 | 8cohort | Not serious | serious | Not serious | Not serious | NA | no | would not reduce effect | no | **Very low** |
| Oral cavity cancer incidence | Wang 2020 | 3cohort | Not serious | Not serious | Not serious | Not serious | NA | no | would not reduce effect | no | **Low** |
| Prostate cancer incidence | Wang 2020 | 9cohort | Not serious | serious | Not serious | serious | NA | no | would not reduce effect | no | **Very low** |
| Skin cancer incidence | Wang 2020 | 3cohort | Not serious | Not serious | Not serious | serious | NA | no | would not reduce effect | no | **Very low** |
| Head and neck cancer overall survival | Van Der Elst 2021 | 7cohort | Not serious | serious | Not serious | Not serious | undetected | no | would not reduce effect | no | **Very low** |
| Glioma survival outcome | Shi 2018 | 6cohort | Not serious | Not serious | Not serious | serious | undetected | no | would not reduce effect | no | **Very low** |
| Breast cancer | Sun 2015 | 11cohort | Not serious | serious | Not serious | serious | undetected | no | would not reduce effect | no | **Very low** |
| Breast cancer recurrence | Xuan Wang 2020 | 7Cohort | Not serious | Not serious | Not serious | Not serious | undetected | no | would not reduce effect | no | **Low** |
| **Mortality outcomes** |  |  |  |  |  |  |  |  |  |  |  |
| All-cause mortality | Wei 2019 | 49cohort | Not serious | Not serious | Not serious | Not serious | strongly suspected | no | would not reduce effect | no | **Very low** |
| CVD mortality | Wei 2019 | 15cohort | Not serious | serious | Not serious | Not serious | strongly suspected | no | would not reduce effect | no | **Very low** |
| Overall survival after transplantation in HSCT | Guillaume 2023 | 8Cohort | Not serious | Not serious | Not serious | serious | strongly suspected | no | would not reduce effect | no | **Very low** |
| All cause mortality with PAD | Scierka 2023 | 4cohort | Not serious | Not serious | Not serious | Not serious | undetected | no | would not reduce effect | no | **Low** |
| All cause mortality in cancer | Wang 2020 | 16cohort | Not serious | Not serious | Not serious | Not serious | undetected | no | would not reduce effect | no | **Low** |
| All cause mortality in lung cancer | Wang 2020 | 3cohort | Not serious | Not serious | Not serious | Not serious | NA | no | would not reduce effect | no | **Low** |
| All cause mortality in breast cancer | Xuan Wang 2020 | 12cohort | Not serious | Not serious | Not serious | Not serious | undetected | no | would not reduce effect | no | **Low** |
| Specific mortality in bladder cancer | Wang 2020 | 2cohort | Not serious | Not serious | Not serious | Not serious | NA | yes | would not reduce effect | no | **Moderate** |
| Specific mortality in colorectum cancer | Wang 2020 | 2cohort | Not serious | Not serious | Not serious | Not serious | NA | no | would not reduce effect | no | **Low** |
| Specific mortality in hematopoietic cancer | Wang 2020 | 2cohort | Not serious | serious | Not serious | Not serious | NA | no | would not reduce effect | no | **Very low** |
| Specific mortality in kidney cancer | Wang 2020 | 2cohort | Not serious | Not serious | Not serious | Not serious | NA | no | would not reduce effect | no | **Very low** |
| Specific mortality in prostate cancer | Wang 2020 | 3cohort | Not serious | Not serious | Not serious | Not serious | NA | no | would not reduce effect | no | **Low** |
| All cause mortality in PCI | Song 2020 | 5cohort | Not serious | Not serious | Not serious | Not serious | undetected | no | would not reduce effect | no | **Low** |
| PSD mortality | Cai 2019 | 14cohort | Not serious | serious | Not serious | Not serious | undetected | no | would not reduce effect | no | **Very low** |
| All cause mortality in HF | Gathright 2017 | 14cohort | Not serious | serious | Not serious | Not serious | strongly suspected | no | would not reduce effect | no | **Very low** |
| Coronary mortality | Wu 2016 | 8cohort | Not serious | serious | Not serious | Not serious | strongly suspected | no | would not reduce effect | no | **Very low** |
| All cause mortality in CABG | Stenman 2016 | 7cohort | Not serious | Not serious | Not serious | Not serious | undetected | no | would not reduce effect | no | **Low** |
| Organ post-transplant mortality | Dew 2016 | 20cohort | Not serious | Not serious | Not serious | Not serious | undetected | no | would not reduce effect | no | **Low** |
| CKD Mortality | Palmer 2013 | 22cohort | Not serious | serious | Not serious | Not serious | strongly suspected | no | would not reduce effect | no | **Very low** |
| All cause mortality with diabetes by self-reports | Hofmann 2013 | 8cohort | Not serious | serious | Not serious | Not serious | undetected | no | would not reduce effect | no | **Very low** |
| All cause mortality with diabetes by clinical interviews supported | Hofmann 2013 | 6cohort | Not serious | serious | Not serious | Not serious | undetected | no | would not reduce effect | no | **Very low** |
| All cause mortality in MI | Meijer 2011 | 17cohort | Not serious | Not serious | Not serious | Not serious | undetected | yes | would not reduce effect | no | **Moderate** |
| Dying in the 2 years after the initial assessment with CHD | Barth 2004 | 7cohort | Not serious | Not serious | Not serious | Not serious | undetected | yes | would not reduce effect | no | **Moderate** |
| Dying in the long-term with CHD | Barth 2004 | 7cohort | Not serious | serious | Not serious | Not serious | undetected | no | would not reduce effect | no | **Very low** |
| **Endocrine/Metabolic outcomes** |  |  |  |  |  |  |  |  |  |  |  |
| Gestational diabetes mellitus | Zhang 2023 | 9cohort | Not serious | Not serious | Not serious | Not serious | undetected | no | would not reduce effect | no | **Low** |
| Diabetic nephropathy | Fang 2022 | 2cross sectional;4cohort | Not serious | Not serious | Not serious | Not serious | undetected | no | would not reduce effect | no | **Low** |
| Metabolic syndrome with cross-sectional studies | Moradi 2021 | 31cross sectional | Not serious | Not serious | Not serious | Not serious | strongly suspected | no | would not reduce effect | no | **Very low** |
| Metabolic syndrome with cohort studies | Moradi 2021 | 18cohort | Not serious | serious | Not serious | Not serious | strongly suspected | no | would not reduce effect | no | **Very low** |
| Type 2 diabetes | Graham 20220 | 15cohort | Not serious | Not serious | Not serious | Not serious | undetected | no | would not reduce effect | no | **Low** |
| Obesity | Mannan 2016 | 9cohort | Not serious | Not serious | Not serious | Not serious | undetected | no | would not reduce effect | no | **Low** |
| **Cardiovascular outcomes** |  |  |  |  |  |  |  |  |  |  |  |
| Incident atrial fibrillation | Fu 2022 | 1case control,8cohort | Not serious | serious | Not serious | serious | undetected | no | would not reduce effect | no | **Very low** |
| Heart failure | Lihuan Cao 2022 | 6cohort | Not serious | Not serious | Not serious | Not serious | NA | no | would not reduce effect | no | **Low** |
| Coronary heart disease | Hongfu Cao 2022 | 26cohort | Not serious | serious | Not serious | Not serious | strongly suspected | no | would not reduce effect | no | **Very low** |
| MACE outcomes with PAD | Abi-Jaoude 2022 | 3cohort | Not serious | serious | Not serious | serious | undetected | no | would not reduce effect | no | **Very low** |
| MALE outcomes with PAD | Abi-Jaoude 2022 | 4cohort | Not serious | serious | Not serious | Not serious | undetected | no | would not reduce effect | no | **Very low** |
| Risk of Readmission in HF | Kewcharoen 2021 | 9cohort;1cross sectional | Not serious | serious | Not serious | Not serious | undetected | no | would not reduce effect | no | **Very low** |
| MACEs after PCI | Song 2020 | 6cohort | Not serious | Not serious | Not serious | Not serious | undetected | yes | would not reduce effect | no | **Moderate** |
| Non-fatal CVD events with type 2 diabetes | Inoue 2020 | 11cohort | Not serious | serious | Not serious | Not serious | undetected | no | would not reduce effect | no | **Very low** |
| Fatal CVD events with type 2 diabetes. | Inoue 2020 | 8cohort | Not serious | Not serious | Not serious | Not serious | undetected | no | would not reduce effect | no | **Low** |
| A composite outcome following PCI | Zhang 2019 | 8cohort | Not serious | Not serious | Not serious | Not serious | undetected | no | would not reduce effect | no | **Low** |
| Recurrent stroke event | Wu 2019 | 6cohort | Not serious | Not serious | Not serious | Not serious | undetected | no | would not reduce effect | no | **Low** |
| Diabetes complication: macrovascular and microvascular | Nouwen 2019 | 3cohort | Not serious | serious | Not serious | Not serious | undetected | no | would not reduce effect | no | **Very low** |
| Ventricular arrhythmias | Fu 2019 | 9cohort | Not serious | serious | Not serious | serious | undetected | no | would not reduce effect | no | **Very low** |
| Coronary artery calcification | Lin 2018 | 6cross sectional, 3cohort,3  case control | Not serious | serious | Not serious | serious | strongly suspected | no | would not reduce effect | no | **Very low** |
| Sudden cardiac death | Shi 2017 | 3cohort,1case-control | Not serious | Not serious | Not serious | Not serious | undetected | no | would not reduce effect | no | **Low** |
| Ventricular tachycardia/ventricular fibrillation | Shi 2017 | 8cohort,1case control | Not serious | Not serious | Not serious | Not serious | undetected | no | would not reduce effect | no | **Low** |
| Myocardial Infarction | Wu 2016 | 9cohort | Not serious | serious | Not serious | Not serious | strongly suspected | no | would not reduce effect | no | **Very low** |
| First-ever stroke | Barlinn 2015 | 28cohort | Not serious | Not serious | Not serious | Not serious | undetected | no | would not reduce effect | no | **Low** |
| Hypertension | Meng 2012 | 9cohort | Not serious | serious | Not serious | Not serious | undetected | no | would not reduce effect | no | **Very low** |
| Cardiac event in MI | Meijer 2011 | 18cohort | Not serious | Not serious | Not serious | Not serious | undetected | no | would not reduce effect | no | **Low** |
| Cardiovascular diseases | Van der Kooy 2007 | 7cohort | Not serious | Not serious | Not serious | Not serious | undetected | no | would not reduce effect | no | **Low** |
| **Digestive outcomes** |  |  |  |  |  |  |  |  |  |  |  |
| Crohn’s disease | Piovani 2023 | 4cohort, 3case-control | Not serious | Not serious | Not serious | serious | undetected | no | would not reduce effect | no | **Very low** |
| Ulcerative colitis | Piovani 2023 | 3cohort,3case-control | Not serious | Not serious | Not serious | Not serious | undetected | no | would not reduce effect | no | **Low** |
| Irritable bowel syndrome | Sibelli 2016 | 8cohort | Not serious | Not serious | Not serious | Not serious | undetected | yes | would not reduce effect | no | **Moderate** |
| **Neurological system outcomes** | |  |  |  |  |  |  |  |  |  |  |
| Postoperative delirium | Diep 2024 | 42cohort | Not serious | Not serious | Not serious | Not serious | undetected | no | would not reduce effect | no | **Low** |
| Motor cognitive risk syndrome | Zhou 2024 | 7 cross-sectional | Not serious | serious | Not serious | serious | undetected | yes | would not reduce effect | no | **Very low** |
| Cognitive score reduction | Mehta 2022 | 29cohort | Not serious | serious | Not serious | Not serious | undetected | no | would not reduce effect | no | **Very low** |
| Mild cognitive impairment | Mehta 2022 | 17cohort | Not serious | serious | Not serious | Not serious | strongly suspected | no | would not reduce effect | no | **Very low** |
| Alzheimer’s disease | Mehta 2022 | 27cohort | Not serious | serious | Not serious | Not serious | undetected | no | would not reduce effect | no | **Very low** |
| Parkinson’s disease | Bareeqa 2022 | 7 cohorts ;8case-control | Not serious | serious | Not serious | serious | NA | yes | would not reduce effect | no | **Very low** |
| Dementia | Santabárbara 2020 | 8cohort | Not serious | serious | Not serious | Not serious | undetected | no | would not reduce effect | no | **Very low** |
| Right hippocampal volume | Santos 2018 | 1cohort; 28cross-sectional | NA | serious | Not serious | Not serious | undetected | no | would not reduce effect | yes | **Low** |
| Left hippocampal volume | Santos 2018 | 1cohort; 28cross-sectional | NA | serious | Not serious | Not serious | undetected | no | would not reduce effect | yes | **Low** |
| **Offspring outcomes** |  |  |  |  |  |  |  |  |  |  |  |
| Childhood asthma in offspring | Jia 2024 | 10cohort | Not serious | Not serious | Not serious | Not serious | undetected | no | would not reduce effect | no | **Low** |
| Depression in offspring (father-child) | Dachew 2023 | 14 cohort, 2 cross-sectional | Not serious | serious | Not serious | serious | undetected | no | would not reduce effect | no | **Very low** |
| Offspring anxiety | Chithiramohan 2023 | 4cohort | Not serious | Not serious | Not serious | Not serious | undetected | no | would not reduce effect | no | **Low** |
| ADHD in offspring | Christaki 2022 | 2case control;6cohort | Not serious | serious | Not serious | Not serious | strongly suspected | no | would not reduce effect | no | **Very low** |
| Apgar score at 1 min | Sun 2021 | 4cohort | Not serious | Not serious | Not serious | Not serious | undetected | no | would not reduce effect | yes | **Low** |
| Low Apgar score at 1 min | Sun 2021 | 3cohort | Not serious | Not serious | Not serious | serious | undetected | no | would not reduce effect | no | **Very low** |
| Apgar score at 5 min | Sun 2021 | 9cohort | Not serious | Not serious | Not serious | Not serious | undetected | no | would not reduce effect | yes | **Low** |
| Low Apgar score at 5 min | Sun 2021 | 4cohort | Not serious | Not serious | Not serious | Not serious | undetected | no | would not reduce effect | no | **Low** |
| Childhood atopic dermatitis | Chen 2021 | 3cohort;1case control | Not serious | serious | Not serious | Not serious | NA | no | would not reduce effect | no | **Very low** |
| Depression in offspring (mother-child) | Tirumalaraju 2020 | 6cohort | Not serious | Not serious | Not serious | Not serious | undetected | no | would not reduce effect | no | **Low** |
| Behavioral problems in children | Cui 2020 | 9cohort | Not serious | Not serious | Not serious | Not serious | undetected | no | would not reduce effect | no | **Low** |
| Emotional problems in children | Cui 2020 | 11cohort | Not serious | Not serious | Not serious | Not serious | undetected | no | would not reduce effect | no | **Low** |
| Social development in children | Cui 2020 | 7cohort | Not serious | serious | Not serious | Not serious | strongly suspected | no | would not reduce effect | no | **Very low** |
| Children’s socio-emotional development | Madigan 2018 | NA | Not serious | Not serious | Not serious | Not serious | undetected | no | would not reduce effect | no | **Low** |
| Child underweight reported | Surkan 2011 | 7 cross sectional,  6case control ;4cohort | Not serious | serious | Not serious | Not serious | undetected | no | would not reduce effect | no | **Very low** |
| Child stunting reported | Surkan 2011 | 7cross-sectional,  1case control ,4cohort | Not serious | serious | Not serious | Not serious | undetected | no | would not reduce effect | no | **Very low** |
| **Dental outcomes** |  |  |  |  |  |  |  |  |  |  |  |
| Dental caries | Cademartori 2018 | 2cross-sectional | Not serious | Not serious | Not serious | Not serious | NA | no | would not reduce effect | no | **Low** |
| Periodontal disease | Cademartori 2018 | 4cross-sectional | Not serious | Not serious | Not serious | serious | NA | no | would not reduce effect | no | **Very low** |
| Tooth loss | Cademartori 2018 | 5cross-sectional | Not serious | Not serious | Not serious | Not serious | NA | no | would not reduce effect | no | **Low** |
| Edentulism | Cademartori 2018 | 4cross-sectional | Not serious | serious | Not serious | serious | NA | no | would not reduce effect | no | **Very low** |
| Periodontitis | Araujo 2016 | 7cross-sectional | Not serious | Not serious | Not serious | serious | undetected | no | would not reduce effect | no | **Very low** |
| Others outcomes | Others outcomes |  |  |  |  |  |  |  |  |  |  |
| Concentrations of CRP | Chen 2024 | 13 cohort | Not serious | serious | Not serious | serious | undetected | no | would not reduce effect | yes | **Very low** |
| Internet addiction | Ye 2023 | 21 cross sectional,1 case-control,1cohort | Not serious | serious | Not serious | Not serious | strongly suspected | no | would not reduce effect | no | **Very low** |
| Pain with acute low back pain | Wong 2022 | 2cohort | Not serious | Not serious | Not serious | serious | undetected | no | would not reduce effect | no | **Very low** |
| Recovery with chronic low back pain | Wong 2022 | 2cohort | Not serious | Not serious | Not serious | serious | undetected | no | would not reduce effect | no | **Very low** |
| Risk of falls | Gambaro 2022 | 2cross sectional,5cohort | Not serious | Not serious | Not serious | serious | undetected | no | would not reduce effect | no | **Very low** |
| Fear of falling | Gambaro 2022 | 2cross sectional,1cohort | Not serious | Not serious | Not serious | Not serious | undetected | yes | would not reduce effect | no | **Moderate** |
| Negative outcomes during  TB treatment. | Ruiz-Grosso 2020 | 2cohort | Not serious | Not serious | Not serious | Not serious | NA | no | would not reduce effect | no | **Low** |
| Medical Errors | Pereira-Lima 2019 | 6cohort,4 cross sectional | Not serious | serious | Not serious | Not serious | undetected | no | would not reduce effect | no | **Very low** |
| Fracture with HR | Wu 2018 | 9cohort | Not serious | serious | Not serious | Not serious | undetected | no | would not reduce effect | no | **Very low** |
| Fracture with RR | Wu 2018 | 7cohort | Not serious | Not serious | Not serious | Not serious | undetected | no | would not reduce effect | no | **Low** |
| Hip bone mineral density | Wu 2018 | 8cohort | Not serious | serious | Not serious | Not serious | undetected | no | would not reduce effect | yes | **Low** |
| Subsequent suicidal behavior | McGinty 2018 | 13cohort | Not serious | Not serious | Not serious | Not serious | undetected | no | would not reduce effect | no | **Low** |
| Frailty with cross-sectional | Soysal 2017 | 4cross sectional | Not serious | Not serious | Not serious | Not serious | undetected | yes | would not reduce effect | no | **Moderate** |
| Frailty with longitudinal | Soysal 2017 | 4cohort | Not serious | serious | Not serious | Not serious | undetected | yes | would not reduce effect | no | **Low** |
| Car crash risk | Hill 2017 | 3 cohort,3 case control | Not serious | serious | Not serious | Not serious | undetected | no | would not reduce effect | no | **Very low** |
| Development of sleep disturbances | Bao 2017 | 11cohort | Not serious | serious | Not serious | Not serious | undetected | no | would not reduce effect | no | **Very low** |
| Persistence of sleep disturbances | Bao 2017 | 7cohort | Not serious | Not serious | Not serious | serious | undetected | no | would not reduce effect | no | **Very low** |
| Worsening of sleep disturbances | Bao 2017 | 2cohort | Not serious | Not serious | Not serious | Not serious | undetected | no | would not reduce effect | no | **Low** |
| Premature ejaculation | Xia 2016 | 2cohort,6cross sectional | Not serious | Not serious | Not serious | Not serious | undetected | no | would not reduce effect | no | **Low** |
| Adult-onset asthma | Gao 2015 | 6cohort | Not serious | Not serious | Not serious | Not serious | undetected | no | would not reduce effect | no | **Low** |
| Sexual dysfunction | Atlantis 2012 | 6cohort | Not serious | serious | Not serious | serious | undetected | no | would not reduce effect | no | **Very low** |

**^1^Evidence classification and definition:**

**High**-- Further research is very unlikely to change our confidence in the estimate of effect.

**Moderate**-- Further research is likely to have an important impact on our confidence in the estimate of effect and may change the estimate

**Low**-- Further research is very likely to have an important impact on our confidence in the estimate of effect and is likely to change the estimate.

**Very low**- Any estimate of effect is very uncertain

**Table S4.** Evidence classification for individual studies included in the umbrella review^1^

| **Outcome** | **Study** | **Sample size** | **Statistical  significance** | **Small-study  effect/excess significant bias** | **95% prediction interval** | **Estimate of  heterogeneity** | **Evidence classification** |
| --- | --- | --- | --- | --- | --- | --- | --- |
| **Cancer outcomes** |  |  |  |  |  |  |  |
| Cancer incidence | Wang 2020 | >1000 | <10^-3^ | >0.05 | Including the null value | very large | III |
| Lung cancer incidence | Wang 2020 | NA | <10^-3^ | NA | Including the null value | very large | III |
| Oral cavity cancer incidence | Wang 2020 | NA | <10^-3^ | NA | Including the null value | not large | III |
| Prostate cancer incidence | Wang 2020 | NA | <0.05 | NA | Including the null value | very large | IV |
| Skin cancer incidence | Wang 2020 | NA | <0.05 | NA | Including the null value | not large | IV |
| Head and neck cancer overall survival | Van Der Elst 2021 | >1000 | <10^-3^ | >0.05 | Including the null value | very large | III |
| Glioma survival outcome | Shi 2018 | <1000 | <0.05 | >0.05 | Including the null value | large | IV |
| Breast cancer | Sun 2015 | >1000 | <10^-3^ | >0.05 | Including the null value | large | III |
| Breast cancer recurrence-free survival | Wang 2020 | >1000 | >0.05 | >0.05 | Including the null value | not large | NS |
| **Mortality outcomes** |  |  |  |  |  |  |  |
| All-cause mortality | Wei 2019 | >1000 | <10^-3^ | <0.05 | Including the null value | not large | III |
| CVD mortality | Wei 2019 | >1000 | <10^-3^ | <0.05 | Including the null value | very large | III |
| Overall survival after transplantation in HSCT | Guillaume 2023 | >1000 | <10^-3^ | <0.05 | Including the null value | not large | III |
| All cause mortality with PAD | Scierka 2023 | >1000 | <0.05 | >0.05 | Including the null value | not large | III |
| All cause mortality in cancer | Wang 2020 | >1000 | <10^-3^ | <0.05 | Including the null value | large | III |
| All cause mortality in lung cancer | Wang 2020 | NA | <10^-3^ | NA | Including the null value | not large | IV |
| All cause mortality in breast cancer | Xuan Wang 2020 | >1000 | >0.05 | >0.05 | Including the null value | not large | NS |
| Specific mortality in bladder cancer | Wang 2020 | NA | <0.05 | NA | Including the null value | not large | IV |
| Specific mortality in colorectum cancer | Wang 2020 | NA | <10^-3^ | NA | Including the null value | large | IV |
| Specific mortality in hematopoietic cancer | Wang 2020 | NA | <10^-3^ | NA | Including the null value | large | IV |
| Specific mortality in kidney cancer | Wang 2020 | NA | <10^-3^ | NA | Including the null value | not large | IV |
| Specific mortality in prostate cancer | Wang 2020 | NA | <10^-3^ | NA | Including the null value | not large | IV |
| All cause mortality in PCI | Song 2020 | >1000 | <10^-3^ | >0.05 | Including the null value | not large | III |
| PSD mortality | Cai 2019 | >1000 | <10^-3^ | >0.05 | Including the null value | very large | III |
| All cause mortality in HF | Gathright 2017 | >1000 | NA | <0.05 | Including the null value | very large | III |
| Coronary mortality | Wu 2016 | >1000 | <10^-3^ | <0.05 | Including the null value | very large | III |
| All cause mortality in CABG | Stenman 2016 | >1000 | >0.05 | >0.05 | Including the null value | large | NS |
| Organ post-transplant mortality | Dew 2016 | >1000 | >0.05 | >0.05 | Including the null value | not large | NS |
| CKD Mortality | Palmer 2013 | >1000 | <0.05 | <0.05 | Including the null value | very large | IV |
| All cause mortality with diabetes by self-reports | Hofmann 2013 | >1000 | <10^-3^ | >0.05 | Including the null value | very large | III |
| All cause mortality with diabetes by clinical interviews supported | Hofmann 2013 | >1000 | <10^-3^ | >0.05 | Including the null value | very large | III |
| All cause mortality in MI | Meijer 2011 | >1000 | <10^-3^ | >0.05 | Including the null value | not large | III |
| Dying in the 2 years after the initial assessment with CHD | Barth 2004 | >1000 | <0.05 | >0.05 | Including the null value | not large | IV |
| Dying in the long-term with CHD | Barth 2004 | >1000 | <0.05 | >0.05 | Including the null value | large | IV |
| **Endocrine/Metabolic outcomes** |  |  |  |  |  |  |  |
| Gestational diabetes mellitus | Zhang 2023 | >1000 | <0.05 | >0.05 | Including the null value | not large | IV |
| Diabetic nephropathy | Fang 2022 | >1000 | <10^-3^ | >0.05 | Including the null value | not large | III |
| Metabolic syndrome with cross-sectional studies | Moradi 2021 | >1000 | <0.05 | >0.05 | Including the null value | large | IV |
| Metabolic syndrome with cohort studies | Moradi 2021 | >1000 | <0.05 | >0.05 | Including the null value | very large | IV |
| Type 2 diabetes | Graham 20220 | >1000 | <0.05 | <0.05 | Including the null value | not large | IV |
| Obesity | Mannan 2016 | >1000 | >0.05 | >0.05 | Including the null value | not large | NS |
| **Cardiovascular outcomes** |  |  |  |  |  |  |  |
| Incident atrial fibrillation | Fu 2022 | >1000 | <0.05 | >0.05 | Including the null value | very large | IV |
| Heart failure | Lihuan Cao 2022 | >1000 | NA | NA | Including the null value | very large | III |
| Coronary heart disease | Hongfu Cao 2022 | >1000 | <10^-3^ | <0.05 | Including the null value | very large | III |
| MACE outcomes with PAD | Abi-Jaoude 2022 | >1000 | <0.05 | <0.05 | Including the null value | very large | IV |
| MALE outcomes with PAD | Abi-Jaoude 2022 | >1000 | <0.05 | <0.05 | Including the null value | large | IV |
| Risk of Readmission in HF | Kewcharoen 2021 | >1000 | <10^-3^ | >0.05 | Including the null value | large | III |
| MACEs after PCI | Song 2020 | >1000 | <10^-3^ | >0.05 | Including the null value | not large | III |
| Non-fatal CVD events with type 2 diabetes | Inoue 2020 | >1000 | <10^-3^ | >0.05 | Including the null value | very large | III |
| Fatal CVD events with type 2 diabetes. | Inoue 2020 | >1000 | >0.05 | >0.05 | Including the null value | not large | NS |
| A composite outcome following PCI | Zhang 2019 | >1000 | <10^-3^ | >0.05 | Including the null value | not large | III |
| Recurrent stroke event | Wu 2019 | >1000 | >0.05 | >0.05 | Including the null value | not large | NS |
| Diabetes complication: macrovascular and microvascular | Nouwen 2019 | >1000 | <10^-3^ | small | Including the null value | very large | III |
| Ventricular arrhythmias | Fu 2019 | >1000 | <10^-3^ | >0.05 | Including the null value | large | III |
| Coronary artery calcification | Lin 2018 | >1000 | <10^-3^ | <0.05 | Including the null value | very large | III |
| Sudden cardiac death | Shi 2017 | >1000 | >0.05 | >0.05 | Including the null value | large | NS |
| Ventricular tachycardia/ventricular fibrillation | Shi 2017 | >1000 | <10^-3^ | >0.05 | Including the null value | not large | III |
| Myocardial Infarction | Wu 2016 | >1000 | <10^-3^ | <0.05 | Including the null value | very large | III |
| First-ever stroke | Barlinn 2015 | >1000 | <0.05 | >0.05 | Including the null value | not large | IV |
| Hypertension | Meng 2012 | >1000 | <0.05 | >0.05 | Including the null value | very large | IV |
| Cardiac event in MI | Meijer 2011 | >1000 | <10^-3^ | >0.05 | Including the null value | not large | III |
| Cardiovascular diseases | Van der Kooy 2007 | >1000 | NA | small | Including the null value | not large | III |
| **Digestive outcomes** |  |  |  |  |  |  |  |
| Crohn’s disease | Piovani 2023 | >1000 | >0.05 | >0.05 | Including the null value | not large | NS |
| Ulcerative colitis | Piovani 2023 | >1000 | <0.05 | >0.05 | Including the null value | not large | IV |
| Irritable bowel syndrome | Sibelli 2016 | >1000 | >0.05 | >0.05 | Including the null value | not large | NS |
| **Neurological system outcomes** |  |  |  |  |  |  |  |
| Postoperative delirium | Diep 2024 | >1000 | <0.05 | >0.05 | Including the null value | not large | IV |
| Motor cognitive risk syndrome | Zhou 2024 | >1000 | <0.05 | >0.05 | Including the null value | very large | IV |
| Cognitive score reduction | Mehta 2022 | >1000 | NA | >0.05 | Including the null value | very large | IV |
| Mild cognitive impairment | Mehta 2022 | >1000 | <0.05 | <0.05 | Including the null value | very large | IV |
| Alzheimer’s disease | Mehta 2022 | >1000 | <0.05 | >0.05 | Including the null value | very large | IV |
| Parkinson’s disease | Bareeqa 2022 | >1000 | <0.05 | NA | Including the null value | very large | IV |
| Dementia | Santabárbara 2020 | >1000 | <0.05 | >0.05 | Including the null value | very large | IV |
| Right hippocampal volume | Santos 2018 | >1000 | <10^-3^ | >0.05 | Including the null value | very large | III |
| Left hippocampal volume | Santos 2018 | >1000 | <0.05 | >0.05 | Including the null value | very large | IV |
| **Offspring outcomes** |  |  |  |  |  |  |  |
| Childhood asthma in offspring | Jia 2024 | >1000 | <10-3 | >0.05 | Including the null value | not large | III |
| Depression in offspring (father-child) | Dachew 2023 | >1000 | <0.05 | >0.05 | Including the null value | very large | IV |
| Offspring anxiety | Chithiramohan 2023 | >1000 | <0.05 | >0.05 | Including the null value | not large | IV |
| ADHD in offspring | Christaki 2022 | >1000 | <10^-3^ | <0.05 | Including the null value | very large | III |
| Apgar score at 1 min | Sun 2021 | >1000 | NA | >0.05 | Including the null value | not large | III |
| Low Apgar score at 1 min | Sun 2021 | >1000 | NA | >0.05 | Including the null value | not large | III |
| Apgar score at 5 min | Sun 2021 | >1000 | NA | >0.05 | Including the null value | not large | III |
| Low Apgar score at 5 min | Sun 2021 | >1000 | NA | >0.05 | Including the null value | not large | III |
| Childhood atopic dermatitis | Chen 2021 | >1000 | <10^-3^ | small | Including the null value | very large | III |
| Depression in offspring (mother-child) | Tirumalaraju 2020 | >1000 | NA | >0.05 | Including the null value | NA | III |
| Behavioral problems in children | Cui 2020 | <1000 | >0.05 | >0.05 | Including the null value | not large | NS |
| Emotional problems in children | Cui 2020 | <1000 | >0.05 | >0.05 | Including the null value | not large | NS |
| Social development in children | Cui 2020 | <1000 | <0.05 | <0.05 | Including the null value | very large | IV |
| Children’s socio-emotional development | Madigan 2018 | >1000 | <10^-3^ | <0.05 | Including the null value | not large | III |
| Child underweight reported | Surkan 2011 | >1000 | NA | >0.05 | Including the null value | not large | III |
| Child stunting reported | Surkan 2011 | >1000 | NA | >0.05 | Including the null value | not large | III |
| **Dental outcomes** |  |  |  |  |  |  |  |
| Dental caries | Cademartori 2018 | >1000 | >0.05 | small | Including the null value | not large | NS |
| Periodontal disease | Cademartori 2018 | >1000 | >0.05 | small | Including the null value | not large | NS |
| Tooth loss | Cademartori 2018 | >1000 | >0.05 | small | Including the null value | not large | NS |
| Edentulism | Cademartori 2018 | >1000 | <0.05 | small | Including the null value | very large | IV |
| Periodontitis | Araujo 2016 | >1000 | <10^-3^ | >0.05 | Including the null value | large | III |
| **Others outcomes** |  |  |  |  |  |  |  |
| Concentrations of CRP | Chen 2024 | >1000 | <10^-3^ | >0.05 | Including the null value | very large | IV |
| Internet addiction | Ye 2023 | >1000 | <10^-3^ | <0.05 | Including the null value | very large | III |
| Pain with acute low back pain | Wong 2022 | <1000 | NA | >0.05 | Including the null value | not large | III |
| Recovery with chronic low back pain | Wong 2022 | >1000 | NA | >0.05 | Including the null value | not large | III |
| Risk of falls | Gambaro 2022 | >1000 | NA | >0.05 | Including the null value | NA | III |
| Fear of falling | Gambaro 2022 | >1000 | NA | >0.05 | Including the null value | NA | III |
| Negative outcomes during  TB treatment. | Ruiz-Grosso 2020 | <1000 | <0.05 | small | Including the null value | not large | IV |
| Medical Errors | Pereira-Lima 2019 | >1000 | <10^-3^ | >0.05 | Including the null value | very large | III |
| Fracture with HR | Wu 2018 | >1000 | <0.05 | >0.05 | Including the null value | large | IV |
| Fracture with RR | Wu 2018 | >1000 | >0.05 | >0.05 | Including the null value | not large | NS |
| Hip bone mineral density | Wu 2018 | >1000 | <0.05 | >0.05 | Including the null value | very large | IV |
| Subsequent suicidal behavior | McGinty 2018 | >1000 | <10^-3^ | >0.05 | Including the null value | large | III |
| Frailty with cross-sectional | Soysal 2017 | >1000 | <10^-3^ | >0.05 | Including the null value | large | III |
| Frailty with longitudinal | Soysal 2017 | >1000 | <10^-3^ | >0.05 | Including the null value | very large | III |
| Car crash risk | Hill 2017 | >1000 | NA | >0.05 | Including the null value | very large | III |
| Development of sleep disturbances | Bao 2017 | >1000 | <10^-3^ | >0.05 | Including the null value | very large | III |
| Persistence of sleep disturbances | Bao 2017 | >1000 | >0.05 | >0.05 | Including the null value | not large | NS |
| Worsening of sleep disturbances | Bao 2017 | >1000 | >0.05 | >0.05 | Including the null value | not large | NS |
| Premature ejaculation | Xia 2016 | >1000 | >0.05 | >0.05 | Including the null value | not large | NS |
| Adult-onset asthma | Gao 2015 | >1000 | <0.05 | >0.05 | Including the null value | not large | IV |
| Sexual dysfunction | Atlantis 2012 | >1000 | <0.05 | >0.05 | Including the null value | large | IV |

^1^Evidence classification criteria

**Class I:** Convincing evidence, >1000 cases (or >20,000 participants for continuous outcomes); statistical significance at P < 10^-6^ (random effects); no evidence of small study effects and excess significance bias; 95% prediction interval excluding null value; no large heterogeneity (I² < 50%).

**Class II:** Highly suggestive evidence, >1000 cases (or >20,000 participants for continuous outcomes); statistical significance at P < 10^-6^ (random effects), and the largest study with a 95% confidence interval excluding the null value.

**Class III:** Suggestive evidence, >1000 cases (or >20,000 participants for continuous outcomes) and statistical significance at P < 0.001.

**Class IV:** Weak evidence, remaining significant associations with P < 0.05.

**NS:** Non-significant, P > 0.05.

Heterogeneity was categorized as not large (I² < 50%), large (I² ≥ 50% but I² ≤ 75%), and very large (I² > 75%)
